# Modelling Incentive Salience in Adolescent Substance Use: The Influence of Substance Cues, Alcohol Expectancies and Socio‐Environmental Factors

**DOI:** 10.1111/adb.70106

**Published:** 2025-11-29

**Authors:** Tommy Gunawan, Michael L. Thomas, Ty Brumback, Duncan B. Clark, David B. Goldston, Kate B. Nooner, Vijay A. Ramchandani, Alejandro D. Meruelo

**Affiliations:** ^1^ Department of Medical and Clinical Psychology Uniformed Services University of the Health Sciences Bethesda Maryland USA; ^2^ Henry M. Jackson Foundation for the Advancement of Military Medicine Bethesda USA; ^3^ Colorado State University Fort Collins Colorado USA; ^4^ Xavier University Cincinnati Ohio USA; ^5^ University of Pittsburgh Pittsburgh Pennsylvania USA; ^6^ Duke University Durham North Carolina USA; ^7^ College of Science & Engineering University of North Carolina Wilmington Wilmington North Carolina USA; ^8^ Human Psychopharmacology Laboratory, Division of Intramural Clinical and Biological Research National Institute on Alcohol Abuse and Alcoholism Bethesda Maryland USA; ^9^ University of California, San Diego La Jolla California USA

**Keywords:** adolescent substance use, confirmatory factor analysis (CFA), exploratory factor analysis (EFA), incentive salience, socio‐environmental moderators, structural equation modelling (SEM)

## Abstract

Alcohol urge regulation, drug urge regulation, motivational attitudes and alcohol expectancies have been linked to substance use, but their combined influence on reward sensitivity and behaviour remains underexplored. This is particularly critical during adolescence and young adulthood, a period of heightened susceptibility to alcohol use and risky behaviours. Using data from the National Consortium on Alcohol and Neurodevelopment in Adolescence (NCANDA), an accelerated longitudinal design that includes all age groups at each time point, this study examined how these factors interact to shape substance use behaviours, including the onset of regular drinking, binge drinking, drug use and sexual intercourse. Exploratory and confirmatory factor analyses (EFA/CFA) utilized data from Years 7 to 9, comprising 430 participants across all age groups, to identify latent incentive‐salience constructs related to urge regulation, motivational attitudes and alcohol expectancies, with a split‐sample approach used to validate the factor structure in a mature cohort. Structural equation modelling (SEM) employed longitudinal data from Year 5 predictors and demographic controls to examine outcomes assessed in Year 6, leveraging the developmental focus of this age range. Covariates included age, sex and socio‐economic status. EFA/CFA confirmed a robust latent structure, and SEM revealed that alcohol urge regulation significantly predicted past‐year binge drinking, while drug urge regulation was negatively associated with total drug use but had no significant effect on alcohol‐related behaviours. Alcohol expectancies and demographic factors, such as older age and male sex, were significantly associated with increased substance use, while socio‐economic status further influenced these outcomes. Findings highlight the critical role of incentive salience in youth substance use and suggest that prevention strategies should target individual risk factors and demographic influences. Early education and supportive policies may reduce early substance use and its associated harms.

## Graphical abstract

Substance use behaviours in adolescents are shaped by personal traits and socio‐environmental factors. Alcohol urge regulation predicted binge drinking, while drug urge regulation was negatively associated with total drug use. Social facilitation expectancies strongly increased binge drinking, highlighting developmental priorities like social integration and peer acceptance. Created in https://BioRender.com.

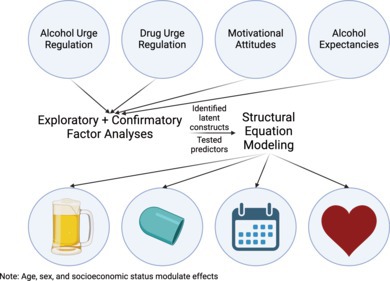

## 1 Introduction

Adolescence and early adulthood are critical phases of neurodevelopment, marked by substantial psychological, social and biological changes that influence behaviour and decision‐making processes [1, 2, 3]. During this period, young people demonstrate increased sensitivity to social contexts [4] and reward‐related cues [5], as well as shifts in their attitudes and priorities regarding social interactions and motivations, including reasons for abstaining from alcohol. These developmental shifts often lead to exploratory behaviours, such as substance use and engagement in potentially risky activities [6, 7]. While these behaviours may reflect normative development, they also carry significant health risks, particularly as individual tendencies interact with broader socio‐environmental factors like social reinforcement and socio‐economic status (SES) [8, 9].

**FIGURE 1:**
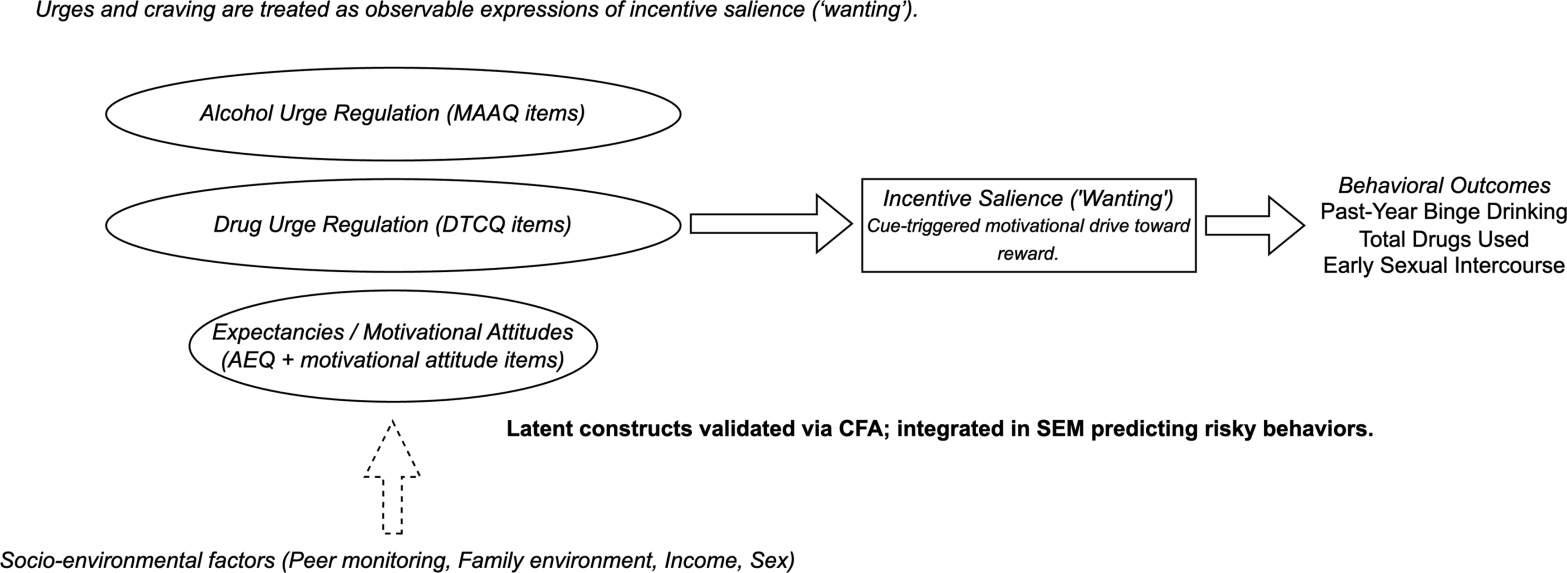
Conceptual model of incentive‐salience components and socio‐environmental moderators predicting adolescent risky behaviours. Urges and craving are treated as observable expressions of incentive salience (‘wanting’). Latent factors representing alcohol urge regulation (MAAQ items), drug urge regulation (DTCQ items) and motivational expectancies/attitudes (AEQ + motivational‐attitude items) converge on the latent construct Incentive Salience, which in turn predicts behavioural outcomes including past‐year binge drinking, total drugs used and early sexual intercourse. Socio‐environmental factors (peer monitoring, family environment, income and sex) act as contextual modifiers. Latent constructs were validated via CFA and integrated within an SEM framework predicting risky behaviours.

Incentive salience provides a mechanistic account of these behaviours. By incentive salience, we mean the cue‐triggered ‘wanting’ that makes reward‐predictive stimuli attention grabbing and approach energizing [10, 11, 12]. This construct is related to but separable from urges and craving: Urges and craving are observable expressions that may vary with context and conscious report, whereas incentive salience refers to the underlying motivational process that can be triggered automatically by cues. Figure 1 depicts this distinction and shows how we operationalize the construct through latent components derived from established instruments: the Drug Taking Confidence Questionnaire (DTCQ; items indexing cue‐ and affect‐elicited urges and regulation of urges) [13, 14, 15, 16], the Motives for Abstaining from Alcohol Questionnaire (MAAQ; items indexing motivational attitudes and reasons not to drink) [17, 18] and the Alcohol Expectancy Questionnaire (AEQ; items indexing positive and negative alcohol outcome expectancies) [19]. These components converge on incentive salience and, in turn, are expected to predict behavioural outcomes including past‐year binge drinking, total drugs used and early sexual intercourse.

This framework is especially relevant in adolescence and early adulthood, when neurodevelopmental changes in frontostriatal circuitry, social salience and affect regulation amplify cue reactivity and goal pursuit [1, 2, 3, 4, 5]. During this window, social settings and emotionally charged contexts can potentiate urges and cravings, strengthen positive expectancies about alcohol and reduce the efficacy of self‐regulatory processes. Accordingly, cues that might be neutral earlier in development can acquire stronger motivational pull, making the downstream influence of incentive salience on risky behaviour more pronounced.

Socio‐environmental factors are integral to this framework because they shape both the expression of incentive salience and the translation of motivation into behaviour. Household income indexes resources and exposures that can buffer or amplify risk and opportunity structures in daily life [20, 21, 22]. Age captures developmental differences in risk‐taking and cue reactivity across adolescence and early adulthood, when neurobiological and social changes dynamically alter sensitivity to reward and control processes [23, 24]. Sex differences—including higher rates of certain substance use behaviours among males—further mark heterogeneity in susceptibility. In addition, proximal contexts such as peer monitoring and family environment can intensify or attenuate cue‐elicited urges, expectancies and motivational attitudes in situ. Accordingly, in our analyses, these variables are modelled as covariates and contextual modifiers of the pathways from latent incentive‐salience components to behavioural outcomes.

We draw on the National Consortium on Alcohol and Neurodevelopment in Adolescence (NCANDA), a prospective, multisite cohort with repeated assessments of alcohol use, related behaviours and psychosocial context. Anchored in the conceptual model in Figure 1, our approach first establishes a theory‐congruent latent structure for incentive‐salience components using exploratory and confirmatory factor analyses and then tests a structural model linking these latent components to risky behaviours with attention to socio‐environmental covariates and temporal ordering between predictors and outcomes [25]. The emphasis is on construct validity and theoretically specified pathways rather than algorithmic prediction.

By elucidating how individual and contextual factors shape incentive salience, this study addresses a gap in the literature and offers practical insights for prevention and intervention. Tailored approaches that target cue‐elicited urges and cravings, modify alcohol expectancies and strengthen abstinence‐supportive motivations within relevant social contexts have the potential to reduce risk and promote positive behavioural outcomes for adolescents and young adults.

Rationale and aims follow directly from this theory and design. The primary aim is to validate a coherent measurement model of incentive salience in adolescence and early adulthood across DTCQ‐, MAAQ‐ and AEQ‐derived indicators and then to evaluate whether these latent components prospectively relate to risky behaviours when accounting for socio‐environmental context. Guided by this framework, we hypothesized that greater alcohol‐related urges, reflecting poorer urge regulation, would predict higher past‐year binge drinking; that greater drug‐related urges would predict a larger number of drug types used; that stronger positive alcohol expectancies and less abstinence‐supportive motivational attitudes would predict higher binge drinking; and that older age and male sex would associate with higher substance use, whereas higher household income would associate with lower risk.

## 2 Method

### 2.1 Participants and Data Collection

The study utilized two distinct, non‐overlapping analytic sets drawn from a larger longitudinal dataset [26]. For the exploratory factor analysis (EFA) and confirmatory factor analysis (CFA), a split‐sample design comprising 430 unique individuals was analysed; together, these participants contributed 21 566 person‐visit observations across later adolescent/young‐adult waves. The split was performed at the participant level to prevent leakage across halves. These data were based on self‐reported assessments of alcohol urge regulation, drug urge regulation, motivational attitudes and alcohol expectancies (Table S1 summarizes EFA/CFA sample characteristics). For the structural equation modelling (SEM), a separate set of *N* = 485 unique individuals was drawn from earlier waves (Table S2 summarizes SEM sample characteristics and visit timing). Importantly, no individual appearing in the EFA/CFA set appeared in the SEM set, ensuring independent evaluation. The EFA/CFA analyses used observation‐level person‐visit records, clustered within participants; the SEM analyses used one record per participant constructed from temporally lagged visits (see below). The SEM analysis included demographic controls such as age, sex and SES.

### 2.2 Analytic Sample and Measures

This study used data from the NCANDA [26], a longitudinal investigation examining the relationship between alcohol use and brain development in participants aged 12–21. NCANDA conducts annual assessments from baseline through Year 9, capturing extensive behavioural, psychological and socio‐environmental data to evaluate substance use and associated risk behaviours. Designed to capture broad geographic representation across the United States, NCANDA recruited participants from multiple regions to ensure inclusion of youth from diverse locations. This collaborative study is carried out across five research sites: UC San Diego, Duke University, the University of Pittsburgh, SRI International and Oregon Health & Science University. For EFA/CFA, we analysed Years 7–9 to maximize mature participant observations needed for split‐sample validation (Table S1). For SEM, predictors and covariates were drawn from Year 5 and outcomes from Year 6 to preserve temporal ordering (Table S2; Figure S1 provides an overview of visit timing and age distributions). The accelerated longitudinal design allowed participants to enter the study at different ages and be assessed annually, providing developmental coverage from adolescence to young adulthood.

### 2.3 Demographic Covariates

The demographic covariates included sex and age at the time of each interview and total household income. Sex was recorded at baseline to account for potential sex differences in substance use and risk behaviours. Age at interview, assessed at each visit, allowed us to control for developmental stage in the analyses, capturing age‐specific effects within the sample's varied age range. Total household income, assessed annually, served as an indicator of SES, controlling for the possible impact of economic background on behavioural outcomes. Total household income was assessed using a modified version of the MacArthur Sociodemographic Questionnaire [27]. For participants living with parents, this measure reflected parental family income, whereas for youths living independently, it represented their individual SES. Annual household income responses were categorized along a socio‐economic scale from lowest to highest: (0) not available (missing), (1) less than $5000, (2) $5000–$11 999, (3) $12 000–$15 999, (4) $16 000–$24 999, (5) $25 000–$34 999, (6) $35 000–$49 999, (7) $50 000–$74 999, (8) $75 000–$99 999, (9) $100 000–$199 999 and (10) $200 000 or greater. Eleven per cent of participants either did not know or chose not to disclose their income data [26].

### 2.4 Predictors (See Supplementary Description for DTCQ/MAAQ/AEQ Question Prompts)

The variables selected for analysis below were chosen based on a missing data threshold of less than 15%, ensuring minimal missingness for robust data analysis.

The *DTCQ* [28] is designed to measure individuals' self‐efficacy or confidence in their ability to resist drug use in various situations. In this study, a more comprehensive subset of DTCQ items was analysed, covering a wider range of scenarios that could challenge one's ability to resist drug use. The items examined were selected based on the least amount of missing data. Specifically, the items examined include Questions 1, 2, 3 and 4 from Set 1; Questions 5 and 6 from Set 2; Questions 1b, 2b, 3b and 4b from Set 3; and Questions 5b and 6b from Set 4. Each item reflects situations that may challenge an individual's drug resistance.

Respondents rate each item on a scale, typically ranging from ‘Not at all confident’ to ‘Extremely confident’, reflecting their confidence in avoiding drug use in specific scenarios. For example, items from Set 1 include scenarios like dealing with emotional stress or social pressure, while Sets 2 and 3 explore managing urges in high‐stress situations or resisting in social gatherings. Higher scores on the DTCQ indicate a stronger perceived ability to resist drug use across various circumstances, shedding light on self‐regulatory capacity and situational resilience.

The *MAAQ* [29] is designed to assess individuals' motivations for choosing not to consume alcohol. The items examined were selected based on the least amount of missing data. In this study, items from the MAAQ include Questions 1, 2, 3 and 4, which cover various personal reasons and motivations for abstaining from alcohol, including health concerns, personal values and social influences.

Each question prompts respondents to rate the degree to which each reason describes their motivation to abstain, typically on a scale from ‘Does not describe me at all’ to ‘Describes me greatly’. For instance, items explore motivations to avoid alcohol for health maintenance or to prevent social or legal consequences. Higher scores reflect stronger motivations for abstaining, providing insights into the personal and social factors that contribute to alcohol avoidance.

The *AEQ* [19] assesses individuals' expectations and beliefs about the effects of alcohol consumption. The items examined were selected based on the least amount of missing data. In this study, a selection of items was used to capture participants' positive or negative anticipations regarding alcohol use. The included questions are 1, 2 and 3 from Set 1; Questions 10, 11, 12, 13 and 14 from Set 2; and Questions 15, 16 and 17 from Set 3, exploring the extent to which individuals believe alcohol might influence their behaviours, emotions or social interactions.

Respondents rate each statement based on how well it reflects their beliefs, typically on a scale from ‘Does not describe me at all’ to ‘Describes me greatly’. Higher scores suggest stronger beliefs in the expected effects of alcohol, which are associated with the likelihood of engaging in alcohol use. This evaluation provides insights into cognitive factors that may predict or moderate alcohol consumption.

### 2.5 Behavioural Outcomes (See Figures S2 and S3 for Visit and Age Distribution)

The behavioural outcomes examined included past‐year binge drinking, onset of regular drinking, total number of drugs used and engagement in sexual intercourse at Year 6.

Past‐year binge drinking reflected the frequency of binge‐drinking episodes (five or more drinks on a single occasion) [30, 31] over the past year.

Onset of regular drinking provided an indicator of when participants began consuming alcohol regularly, defined as drinking at least once a week.

Total number of drug types used, captured the cumulative count of different substances participants reported using, excluding alcohol, marijuana and nicotine, thus reflecting the diversity of substance use.

Additionally, engagement in sexual intercourse, served as an indicator of sexual risk‐taking, representing broader tendencies towards risk behaviours [32].

### 2.6 EFA

To uncover the latent constructs underlying incentive salience, we conducted an EFA using maximum likelihood (ML) estimation with a Varimax rotation, applying input variables provided in Table S1. The EFA was performed on a randomly selected participant‐level half sample of the 21 566 person‐visit observations from Years 7 to 9 of NCANDA, allowing us to explore factor structures without overlap with the CFA half. ML estimation was chosen following checks for approximate normality. We systematically tested models with two to seven factors, evaluating model fit using AIC, BIC, RMSEA and CFI, in addition to parallel analysis and the scree plot of eigenvalues to determine the optimal factor structure. The scree plot showed an inflection point after six factors, supporting the selection of this model. Factor loadings above 0.3 were deemed significant, and items with substantial cross‐loadings or low communalities were carefully assessed for retention. This analysis yielded a six‐factor model representing distinct but interconnected incentive‐related constructs, carried forward for confirmatory testing.

### 2.7 CFA

Building on the EFA results, we conducted a CFA on the held‐out participant‐level half sample of the Years 7–9 observations to validate the six‐factor structure. The CFA used ML estimation with full‐information ML (FIML) to handle missing data, implemented in the lavaan package (Version 0.6‐19) [33]. As sensitivity checks for non‐independence and uncertainty, we computed participant‐cluster‐robust standard errors and, separately, non‐parametric bootstrap intervals (2000 draws) for factor loadings. We assessed model adequacy through CFI, TLI, RMSEA (90% CI) and SRMR [34] and made iterative, theory‐consistent adjustments when indicated, focusing on improving fit by addressing items with low factor loadings or substantial residual correlations; items were restricted to load on a single factor.

### 2.8 SEM Construction

Following confirmation of the factor structure through CFA, which we henceforth used as our measurement model, we specified a theory‐guided, time‐lagged SEM to investigate the roles of the latent incentive‐salience factors—including alcohol urge regulation, drug urge regulation, motivational attitudes and alcohol expectancies—in predicting substance use behaviours (e.g., past‐year binge drinking) and other risky behaviours (e.g., sexual intercourse). To ensure temporal precedence and independence from the EFA/CFA analyses, predictors and covariates were taken from Year 5 and outcomes from Year 6. We emphasized developmental interpretability and parsimony; we therefore did not estimate growth or random‐intercept cross‐lagged models, which are reserved for future work centred on developmental change. Age, sex and household income were included as covariates. Small positive constraints were applied to observed variable residual variances where necessary to stabilize estimation and prevent inadmissible solutions. Models were estimated with the NLMINB optimizer; diagnostics guided limited, theory‐consistent residual covariance adjustments and variance constraints. Models were estimated using ML with robust (Huber–White) standard errors and a mean/variance adjusted test statistic (MLR) with FIML for missing data, applying Yuan–Bentler corrections [35, 36] (Std.lv = TRUE; fixed.x = FALSE).

### 2.9 Units of Analysis and Non‐Independence

The EFA and CFA phases treated the unit of analysis as person‐visit observations within participant‐level split halves (Table S1), whereas the SEM phase used one record per participant constructed from Year 5 predictors/covariates and Year 6 outcomes (Table S2). To address potential non‐independence from repeated measures in CFA, we conducted sensitivity analyses using participant‐cluster‐robust standard errors and, separately, a two‐level CFA on a reduced indicator set. For SEM, models were estimated with FIML under MLR with Yuan–Bentler robust corrections (Std.lv = TRUE; fixed.x = FALSE).

### 2.10 Model Fit Evaluation and Interpretation

Fit indices were rigorously evaluated to assess model adequacy, following conventional guidance [25, 34]: CFI and TLI ≥ 0.95 indicate close fit and ≥ 0.90 acceptable fit; RMSEA ≤ 0.06 indicates close fit and ≤ 0.08 acceptable fit; SRMR ≤ 0.08 indicates acceptable fit. We report *χ* ^2^ statistics for completeness while interpreting fit indices in light of model complexity and indicator count. Where thresholds were modestly relaxed (e.g., CFA RMSEA ≈ 0.07), we justify this based on the number of items, construct breadth and parsimony and verify that substantive inferences are consistent across robustness checks (cluster‐robust standard errors and non‐parametric bootstrap). Standardized estimates are used to interpret the strength and direction of relations between latent incentive‐salience constructs—alcohol urge regulation, drug urge regulation, motivational attitudes and alcohol expectancies—and behavioural outcomes; SEM fit and inferences rely on MLR with Yuan–Bentler robust corrections.

### 2.11 Preregistration

This is a secondary analysis of a publicly available longitudinal cohort; no preregistration was conducted, but the analytic plan was specified a priori.

## 3 Results

The results of this study are organized into three main sections: (1) EFA, (2) CFA and (3) SEM. Each section details the steps taken to identify and validate the latent constructs underlying incentive salience, focusing on alcohol urge regulation, drug urge regulation, motivational attitudes and specific alcohol expectancies. These analyses examine how these latent factors, essential to understanding the drive for incentive salience, shape substance use and related behaviours.

### 3.1 EFA

**FIGURE 2:**
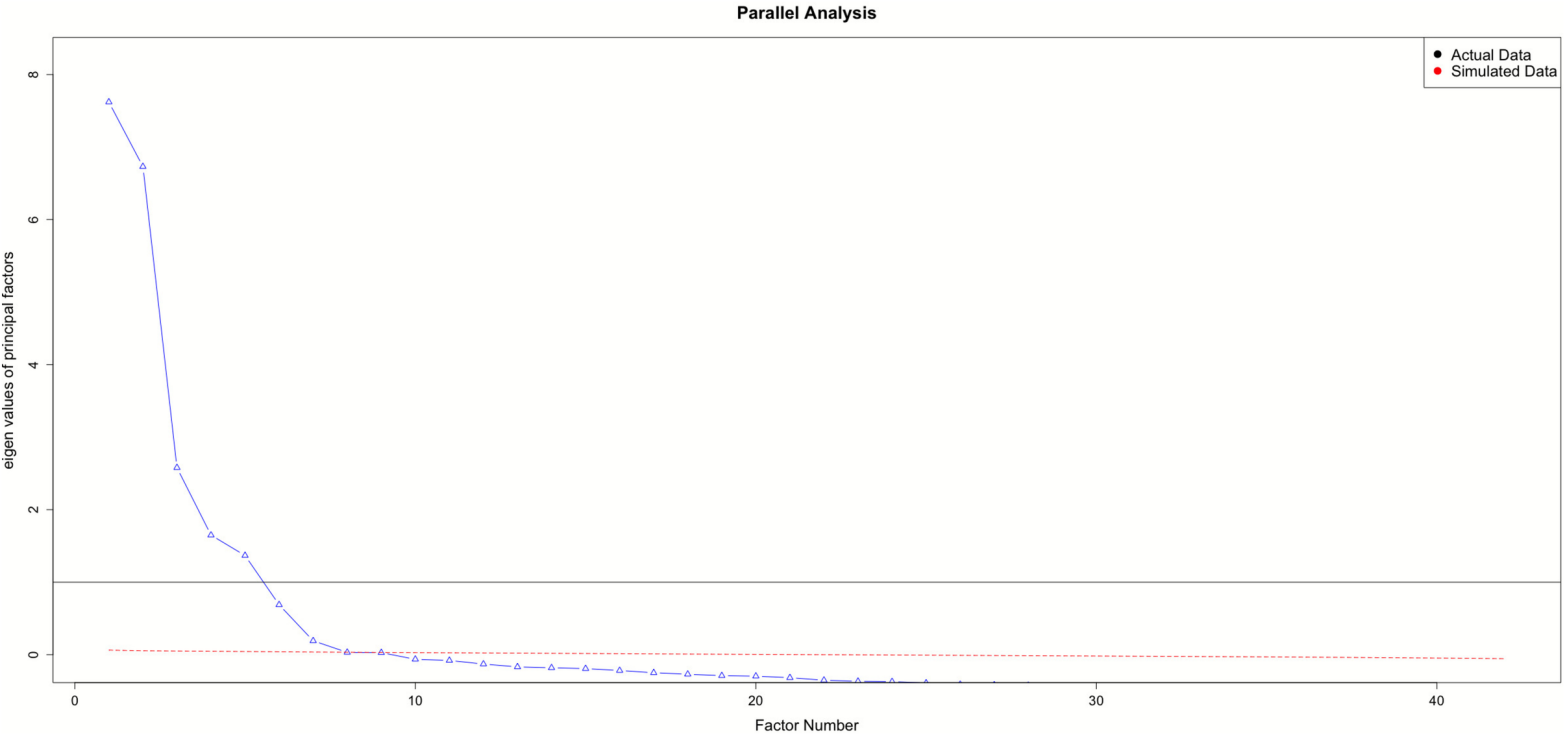
Parallel analysis scree plot. The scree plot showed an inflection point (i.e., elbow) after six factors, supporting the selection of the six‐factor model.

To identify the underlying factor structure, we conducted an EFA with 10 783 participant observations (representing 418 unique individuals) [Tables S3–S9]. The analysis indicated that a six‐factor model was suitable, confirmed by both a parallel analysis and a scree plot (Figure 2), with improvements in model fit reflected in RMSEA (0.069), RMSR (0.03) and BIC values (59 095.58). The six‐factor model explained 54% of the variance, with cumulative variance proportions for individual factors as follows: Factor 1 (31%), Factor 2 (25%), Factor 3 (20%), Factor 4 (9%), Factor 5 (8%) and Factor 6 (7%). Factors were orthogonal, and therefore, there was no correlation matrix for the six factors.

Factor loadings revealed distinct item groupings, uncovering latent constructs associated with incentive salience, including factors relating to alcohol urge regulation, drug urge regulation, motivational attitudes and alcohol expectancies. Communalities ranged from 0.21 to 0.83, suggesting a moderate to high degree of shared variance within factors. Correlations between the factors, particularly among those related to alcohol urge regulation, drug urge regulation and motivational attitudes, provided deeper insight into the dimensions of incentive salience, which informed the construction of the CFA model to further validate this structure.

The factor score reliability measures were strong, with minimum correlations for factors ranging from 0.53 to 0.89 and factor loadings exceeding 0.60 in most cases, indicating robust factor adequacy.

### 3.2 CFA

Following the EFA, we conducted a CFA to validate the refined six‐factor structure encompassing latent constructs related to incentive salience, including alcohol urge regulation (Factor 1), drug urge regulation (Factor 2), motivational attitudes (Factor 3), social facilitation alcohol expectancies (Factor 4) and arousal and relaxation alcohol expectancies (Factor 5) (Tables S10–S13). However, Factor 6 was removed due to the lack of convergence, and EFA factor loadings AEQ1.4–1.7 were excluded from the CFA model to ensure model convergence and fit.

The baseline robust CFA (MLR, no clustering) indicated acceptable fit (scaled *χ* ^2^(310) = 25 347.56, *p* < 0.001, CFI = 0.906, TLI = 0.885, RMSEA = 0.061, 90% CI 0.061–0.070, SRMR = 0.051; Table S7). A Bollen–Stine bootstrap also rejected exact fit (*p* < 0.001), and bootstrap SEs/CIs yielded substantively similar loadings (Tables S14 and S15).

To address non‐independence from repeated observations, we ran a subject‐cluster‐robust CFA (scaled–shifted test). Robust fit was comparable or slightly improved (CFI = 0.921, TLI = 0.911, RMSEA = 0.063, SRMR = 0.049), and standardized loadings were materially unchanged (Table S14). As a complementary check, a two‐level CFA on a reduced indicator set showed broadly similar loading patterns at within‐ and between‐person levels (Table S15).

Standardized factor loadings ranged from ~0.49 to ~0.92 (all *p* < 0.001), reinforcing the robustness of the factor structure. Notable covariances were observed among latent factors, particularly between alcohol urge regulation (Factor 1) and drug urge regulation (Factor 2), as well as motivational attitudes and alcohol expectancies, aligning with the incentive‐salience framework. Together, the robustness checks (cluster‐robust, bootstrap/Bollen–Stine and two‐level CFA) support the same substantive conclusions.

### 3.3 SEM

**FIGURE 3:**
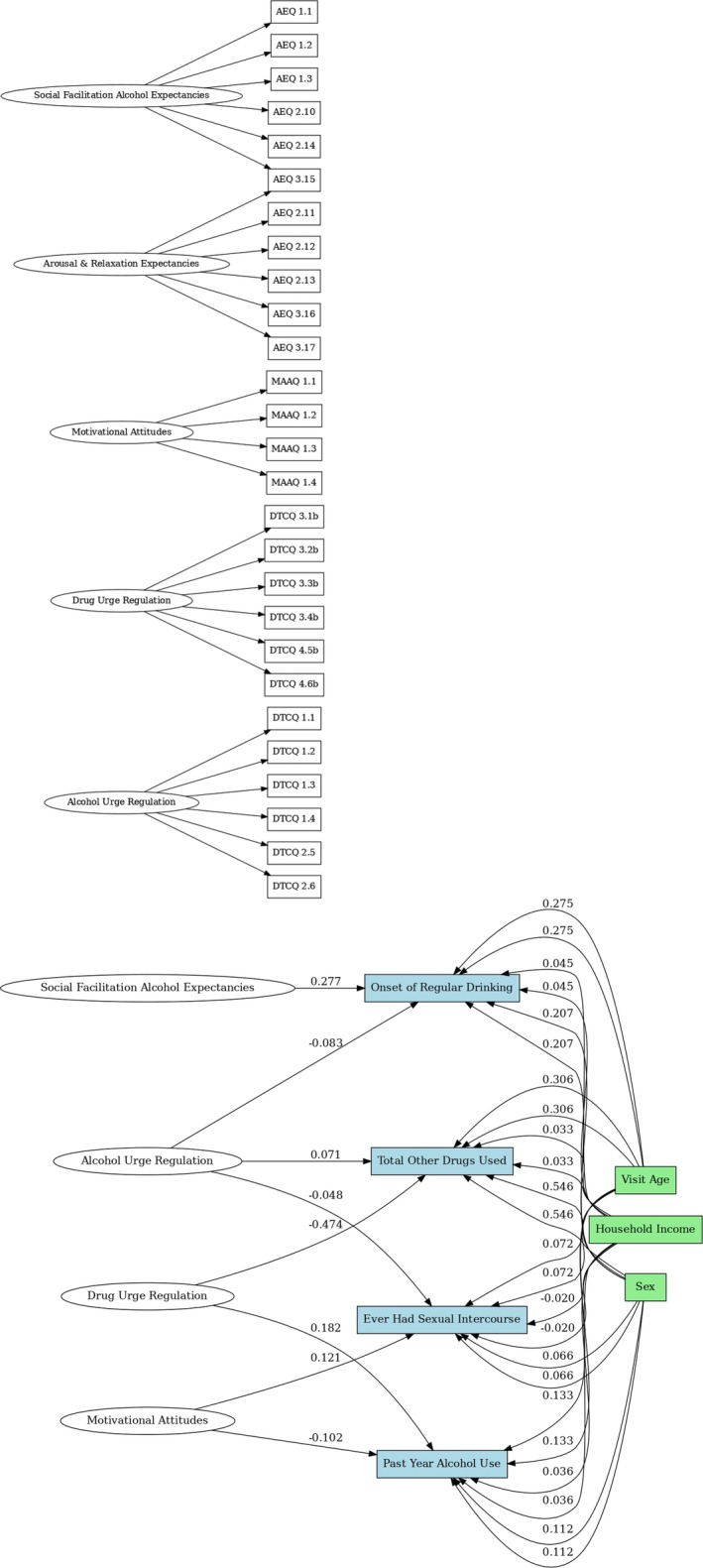
Path diagram for structural equation model. Measurement and factors‐to‐outcomes models. The measurement model illustrates the latent constructs (Factors 1–4) and their relationships with observed variables. The factors include Alcohol Urge Regulation (Factor 1), Drug Urge Regulation (Factor 2), Motivational Attitudes (Factor 3) and Social Facilitation Alcohol Expectancies (Factor 4). The factors‐to‐outcomes model depicts the pathways from these latent constructs to key outcomes, including Onset of Regular Drinking, Past‐Year Alcohol Use, Total Other Drugs Used and Ever Having Had Sexual Intercourse, while controlling for covariates such as Sex, Visit Age and Household Income. Arousal & Relaxation Expectancies (Factor 5) has been removed from this model as it did not significantly predict any of the outcomes, highlighting its limited role in explaining the variance in these behavioural outcomes within this sample.

The SEM model (Table 1), utilizing latent factors validated through CFA, examined predictive relationships between substance use behaviours and underlying constructs such as alcohol urge regulation, drug urge regulation, motivational attitudes and alcohol expectancies (Table S1). These central latent constructs capture dimensions of incentive salience and were analysed in relation to substance use outcomes. To improve model fit and convergence, Factor 2 for the onset of regular drinking and Factors 2 and 3 for ever having had sexual intercourse were removed from the analysis. Notably, the model leveraged items from Year 5 to predict Year 6 outcomes, a key feature of the longitudinal design that allows for stronger causal inferences by temporally associating predictors and outcomes. Socio‐environmental covariates—age, sex and household income—were included to assess their influence on these pathways (Figure 3). Cross‐loadings for Factors 4 and 5 from the CFA were introduced to improve the model fit.

**TABLE 1:** SEM model findings (*N* = 485).

Outcome variable	Predictor variable	Estimate	SE	*z* value	*p*(> |*z*|)	Std.lv	Std.all
Onset of regular drinking	Factor 1	−0.083	0.055	−1.521	0.128	−0.072	−0.066
Onset of regular drinking	Factor 3	0.131	0.128	1.029	0.304	0.055	0.05
Onset of regular drinking	Factor 4	0.277	0.081	3.421	0.001	0.174	0.159
Onset of regular drinking	Visit age	0.275	0.031	8.926	0	0.275	0.388
Onset of regular drinking	Total household income	0.045	0.019	2.326	0.02	0.045	0.101
Onset of regular drinking	Sex	0.207	0.091	2.279	0.023	0.207	0.094
Total other drugs used	Factor 1	0.071	0.111	0.638	0.524	0.061	0.034
Total other drugs used	Factor 2	−0.474	0.113	−4.209	0	−0.398	−0.22
Total other drugs used	Factor 3	−0.415	0.216	−1.92	0.055	−0.175	−0.097
Total other drugs used	Factor 4	0.245	0.135	1.815	0.07	0.154	0.085
Total other drugs used	Visit age	0.306	0.052	5.918	0	0.306	0.263
Total other drugs used	Total household income	0.033	0.032	1.021	0.307	0.033	0.045
Total other drugs used	Sex	0.546	0.153	3.576	0	0.546	0.151
Ever had sexual intercourse?	Factor 1	−0.048	0.034	−1.409	0.159	−0.042	−0.065
Ever had sexual intercourse?	Factor 4	−0.023	0.05	−0.463	0.643	−0.015	−0.023
Ever had sexual intercourse?	Visit age	0.072	0.019	3.745	0	0.072	0.174
Ever had sexual intercourse?	Total household income	−0.02	0.012	−1.663	0.096	−0.02	−0.077
Ever had sexual intercourse?	Sex	0.066	0.057	1.163	0.245	0.066	0.051
Past‐year binge	Factor 1	−0.182	0.062	−2.962	0.003	−0.158	−0.157
Past‐year binge	Factor 2	0.063	0.061	1.024	0.306	0.052	0.052
Past‐year binge	Factor 3	−0.071	0.119	−0.596	0.551	−0.03	−0.03
Past‐year binge	Factor 4	0.456	0.078	5.847	0	0.287	0.286
Past‐year binge	Visit age	0.133	0.029	4.598	0	0.133	0.205
Past‐year binge	Total household income	0.036	0.018	1.992	0.046	0.036	0.089
Past‐year binge	Sex	0.112	0.085	1.317	0.188	0.112	0.056

*Note:* This table summarizes the findings from the SEM analysis examining predictors of four key substance use outcomes: onset of regular drinking, total other drugs used, past‐year binge drinking and having ever had sexual intercourse. Predictor variables include latent factors (Factors 1–4), demographic covariates (visit age, household income and sex) and other contextual variables. Reported statistics include unstandardized estimates, standard errors (SE), *z* values, *p* values and standardized estimates (Std.lv for latent variables and Std.all for all observed variables). Significant predictors are highlighted by *p* values less than 0.05, such as Factor 4's strong association with past‐year binge drinking (Estimate = 0.456, *p* < 0.001). Additionally, demographic variables such as visit age and total household income show notable effects across multiple outcomes. These findings underscore the complex interplay of psychological, behavioural and demographic factors in shaping substance use behaviours.

Abbreviations: SE = standard error; Std.all = standardized loading for both latent and observed variables.

The model demonstrated an acceptable fit, with a chi‐square statistic of 1340.549 (df = 487, *p* < 0.001), CFI of 0.877, TLI of 0.859 and RMSEA of 0.060 (90% CI 0.056–0.064), indicating alignment with observed data. An SRMR of 0.058 further supported the model fit. Findings revealed that alcohol urge regulation (Factor 1) significantly predicted past‐year binge drinking, with higher levels associated with a higher likelihood of past‐year binge drinking. In contrast, drug urge regulation (Factor 2) was inversely associated with total drug use but did not significantly influence binge drinking or age of alcohol initiation. Social facilitation alcohol expectancies (Factor 4) showed a strong positive association with increased past‐year binge drinking. Arousal & Relaxation Expectancies (Factor 5) did not significantly predict any of the outcomes and therefore were removed from the SEM for simplicity of presentation.

To assess robustness and the independence of observations in the SEM, we estimated the model with FIML under MLR and Yuan–Bentler corrections (Std.lv = TRUE; fixed.x = FALSE). Robust fit was acceptable (scaled *χ* ^2^(488) = 1064.65, *p* < 0.001, CFI = 0.883, TLI = 0.866, RMSEA = 0.069, 90% CI 0.061–0.076, SRMR = 0.071, *N* = 664), and inferences for all structural paths were materially unchanged relative to the baseline specification (Table S16). Because each participant contributes a single Year 5 → Year 6 record in the SEM, residual non‐independence from repeated visits is minimized by design; nevertheless, results were stable when using robust test statistics and standard errors. As an additional sensitivity analysis, we attempted a WLSMV specification treating the question, ‘Ever had sexual intercourse?’ as ordered; this model was unstable (near‐unit polyserial correlations and very small effective pairwise *N*) and did not converge, so primary conclusions are based on the MLR/FIML model, for which effect directions and magnitudes were consistent across robustness checks.

Among the socio‐environmental factors, older age consistently correlated with higher substance use, and higher household income appeared protective, particularly against binge drinking. In summary, these results emphasize the interplay between alcohol urge regulation, drug urge regulation, motivational attitudes, alcohol expectancies and socio‐environmental influences, offering a nuanced understanding of the pathways leading to substance use among adolescents and young adults. These insights into alcohol/drug urge regulation, attitudes and expectancies deepen our grasp of factors shaping youth behaviour in substance use contexts.

## 4 Discussion

This study aimed to identify and validate latent structures representing the underlying incentive salience associated with alcohol urge regulation, drug urge regulation, motivational attitudes and alcohol expectancies and to examine their effects on substance use behaviours among adolescents and young adults. Through a sequential analysis—including EFA, CFA and SEM—three key findings emerged. These findings provide deeper insight into how latent factors tied to incentive salience, alongside socio‐environmental influences, contribute to substance use outcomes. Because our primary objective was rigorous measurement validation to ground subsequent hypothesis testing, we emphasized EFA/CFA before SEM, with multimodal extensions (e.g., neuroimaging integration) intentionally reserved for future work built on this validated measurement base.

### 4.1 EFA Findings: Core Latent Constructs Underlying Incentive Salience

The EFA revealed a robust six‐factor structure capturing the core elements of incentive salience, including alcohol urge regulation (Factor 1), drug urge regulation (Factor 2), motivational attitudes (Factor 3) and social facilitation alcohol expectancies (Factor 4). The factor structure provides a comprehensive framework to understand the complex traits and motivations associated with youth behaviour and incentive salience, collectively influencing substance use risk.

### 4.2 CFA Findings: Validation of Factor Structure and Interrelationships

The CFA validated the six‐factor model identified in the EFA, demonstrating strong alignment with the hypothesized structure. Significant associations were observed among the factors, particularly between alcohol urge regulation (Factor 1) and drug urge regulation (Factor 2), as well as between motivational attitudes (Factor 3) and social facilitation alcohol expectancies (Factor 4). These associations suggest that alcohol and drug urge regulation collectively heighten susceptibility to incentive‐driven behaviours, while motivational attitudes and alcohol expectancies influence cognitive and emotional responses to potential rewards, such as substance use.

The validated factor structure provides a robust framework for guiding future research and interventions. For instance, the strong interrelationship between alcohol and drug urge regulation highlights the need for integrated approaches targeting multiple substances rather than focusing on single‐substance interventions. Additionally, the association between motivational attitudes and alcohol expectancies suggests that interventions could benefit from addressing cognitive processes, such as reframing expectations around substance use rewards or enhancing social coping strategies, to reduce the appeal of risky behaviours. In practical terms, the pattern of associations provides a direct line from measurement to intervention. Skills that target craving and cue reactivity may mitigate the influence of alcohol urge regulation, expectancy‐challenge and cognitive‐behavioural modules that recalibrate anticipated social rewards may attenuate the impact of alcohol expectancies and values‐based goal setting with skills training may redirect motivational attitudes towards adaptive, nonsubstance rewards.

### 4.3 SEM Findings: The Role of Socio‐Environmental Influences in Incentive Salience

The SEM analysis underscores the intricate interplay between personal traits and socio‐environmental factors in shaping substance use behaviours among adolescents and young adults. Alcohol urge regulation (Factor 1) was a significant predictor of past‐year binge drinking. This suggests that coping‐related urges and situational triggers for alcohol increase susceptibility to more intensive use patterns. In contrast, drug urge regulation (Factor 2) showed a nuanced effect, with a negative association with total drug use but no significant influence on alcohol‐related behaviours. These distinctions highlight how different coping mechanisms and substance‐specific urges uniquely impact substance use.

The finding that social facilitation alcohol expectancies (Factor 4) strongly predict increased past‐year binge drinking is consistent with a robust body of literature on the role of alcohol expectancies in driving drinking behaviours. Research has repeatedly shown that positive alcohol expectancies—beliefs about the social and emotional benefits of drinking—are significant predictors of initiation, frequency and intensity of alcohol use, particularly among adolescents and young adults [37]. Social facilitation expectancies, specifically, are particularly salient in this age group, as they align with developmental priorities like social integration, peer acceptance and identity formation [38].

Studies suggest that these expectancies not only predict binge drinking but may also mediate the relationship between peer influence and alcohol use, amplifying risky behaviours in social contexts [39]. Additionally, heightened expectancies of social enhancement may create a feedback loop, where positive reinforcement from social drinking experiences reinforces the expectancy and leads to continued heavy drinking. This underscores the importance of targeting alcohol expectancies in prevention and intervention programmes. Cognitive‐behavioural approaches that challenge these beliefs and provide alternative coping strategies may reduce the influence of such expectancies on binge‐drinking tendencies. Future research could further investigate how specific types of alcohol expectancies, such as those related to social facilitation, interact with environmental and individual risk factors to shape alcohol use trajectories. Taken together with our SEM results, these data indicate that modifying expectancies and strengthening context‐specific self‐regulation skills are plausible, testable levers for reducing binge‐drinking risk during this developmental window.

The protective effects of higher SES identified in this study align with existing literature emphasizing the role of SES in mitigating substance use behaviours. Higher household income, as observed, has been consistently associated with reduced risk of substance use and related problems, potentially through increased access to resources such as education, healthcare and extracurricular activities, which serve as protective factors [40]. Furthermore, higher SES often correlates with greater parental monitoring and structured environments, which have been shown to deter risky behaviours like binge drinking [41]. However, some studies have identified nuanced relationships where high SES, particularly in affluent communities, may also increase risk for substance use through heightened social pressures or cultural norms around alcohol and drug use [42]. This highlights the importance of considering context and specific mechanisms when interpreting SES effects. Future research should further explore the protective and potentially risk‐enhancing roles of SES across diverse populations to clarify these dynamics.

These findings underscore the importance of considering both individual and environmental factors in substance use interventions targeting youth, aligning with the limited but growing body of literature on this topic [43, 44, 45, 46, 47]. The significant roles of alcohol/drug urge regulation and alcohol expectancies underscore the potential for interventions targeting these traits to be more effective if they also account for socio‐environmental contexts, such as family support and financial stability. By understanding how personal characteristics interact with environmental factors, this model provides actionable insights for developing tailored interventions that address the multifaceted nature of substance use risks during critical developmental stages.

The present analyses were designed to validate measurement structure (EFA/CFA) and estimate theory‐guided pathways (SEM) using behavioural/survey data as a first step. Neuroimaging and other multimodal extensions were omitted by design to avoid conflating construct validation with modality‐specific effects, and planned next studies will integrate imaging to test whether the validated factors map onto neural circuits implicated in incentive salience.

### 4.4 Limitations

Although this study provides valuable insights, several limitations should be considered to contextualize the findings. First, the generalizability of results is limited by the sample's demographic characteristics. NCANDA participants tend to reflect higher SES and less racial and ethnic diversity compared to broader populations, such as those represented in the ABCD study [48]. This may influence the applicability of findings to populations with greater socio‐economic adversity, where different environmental stressors or access to resources could alter the relationships between incentive‐salience constructs and substance use behaviours. For example, youth from lower income backgrounds may face unique contextual pressures, such as financial insecurity or limited access to supportive interventions, that heighten susceptibility to risky behaviours in ways not captured here. Future research with more representative samples would enhance the external validity of these findings.

Second, reliance on self‐reported measures for substance use behaviours and personality traits introduces potential biases, such as social desirability or recall inaccuracies. For instance, participants may underreport behaviours like binge drinking or drug use due to stigma or overestimate personality traits associated with reward seeking. Incorporating objective assessments, such as biological markers of substance use, or external informant reports in future studies could reduce these biases and improve measurement reliability.

Third, it is important to acknowledge the correlational and naturalistic nature of the data, which limits the ability to infer causality. Although the analyses identify associations between self‐reported substance use behaviours, personality traits and other variables of interest, the directionality or underlying mechanisms of these relationships cannot be determined. Unmeasured confounding variables or bidirectional effects may also contribute to observed patterns. Follow‐up research using experimental designs could provide stronger evidence for causal pathways and clarify the temporal dynamics of these associations.

In the measurement phase (EFA/CFA), repeated person‐visit observations introduce potential within‐person dependence and the possibility that within‐ and between‐person structures diverge. We probed this by estimating participant‐cluster‐robust standard errors and, separately, a two‐level CFA on a reduced indicator set; inferences were materially unchanged. The primary SEM was specified at the between‐person level using a single record per participant (Year 5 predictors/covariates → Year 6 outcomes) and was estimated via FIML under MLR with Yuan–Bentler robust corrections (Std.lv = TRUE; fixed.x = FALSE). While we did not fit a multilevel SEM, concordance between the validated measurement model and the SEM results, together with the CFA sensitivities, mitigates concern that nonmodelled nesting biased the substantive conclusions; nonetheless, because factor structures can differ across levels, multilevel extensions are a priority for future work [49].

Finally, while this study included key socio‐environmental covariates like age and household income, it did not examine other potentially impactful factors, such as school climate, neighbourhood characteristics or mental health status. These factors might provide additional explanatory power, particularly in understanding variability in substance use behaviours across different social and developmental contexts. Addressing these gaps in future research would yield a more comprehensive understanding of the interplay between individual and contextual factors in shaping youth substance use trajectories. In addition, neuroimaging modalities were not included here by design, as the current report prioritized construct validation; a companion analysis is planned to test multimodal extensions using the validated factor structure.

## 5 Conclusion

In conclusion, this study offers a nuanced framework for understanding the incentive salience underlying youth behaviours, detailing how latent factors, such as responses to alcohol/drug urge regulation and alcohol expectancies, interact with socio‐environmental influences to impact substance use. By establishing these constructs and clarifying their interrelationships, this work lays a foundation for interventions that are tailored to the motivational and environmental contexts of adolescents and young adults vulnerable to substance use. By linking validated latent constructs to specific, modifiable targets—craving/cue‐reactivity skills, expectancy‐challenge/cognitive‐behavioural modules and values‐based skills training—these results offer a concrete pathway from measurement to mechanism‐informed intervention.

## Author Contributions

**Tommy Gunawan:** conceptualization, methodology, formal analysis, visualization, writing – review and editing. **Michael L. Thomas:** methodology, formal analysis, supervision, writing – review and editing. **Ty Brumback:** methodology, writing – review and editing. **Duncan B. Clark:** writing – review and editing, funding acquisition, resources. **David B. Goldston:** writing – review and editing, funding acquisition, resources. **Kate B. Nooner:** writing – review and editing, funding acquisition, resources. **Vijay A. Ramchandani:** writing – review and editing, supervision, funding acquisition. **Alejandro D. Meruelo:** conceptualization, formal analysis, writing – original draft, writing – review and editing, funding acquisition.

## Funding

This work was supported by the National Institute on Alcohol Abuse and Alcoholism (NIAAA) through a grant awarded to Alejandro D. Meruelo, MD, PhD (K23 AA026869); the Division of Intramural Clinical and Biological Research, NIAAA, NIH; and the NIAAA through funding for the National Consortium on Alcohol and Neurodevelopment in Adolescence (NCANDA; Grants AA021697, AA021695, AA021692, AA021696, AA021681, AA021690 and AA021691).

## Ethics Statement

This study is a secondary analysis of de‐identified data from the National Consortium on Alcohol and Neurodevelopment in Adolescence (NCANDA). The original NCANDA study was approved by the Institutional Review Boards (IRBs) at all participating institutions, including the University of California San Diego, Duke University, the University of Pittsburgh, SRI International and Oregon Health & Science University. All participants provided written informed consent or assent, and parental consent was obtained for participants under the age of 18 at the time of enrolment. This secondary analysis was deemed exempt from further IRB review under federal regulations for research involving de‐identified data (45 CFR 46.104).

## Conflicts of Interest

The authors declare no conflicts of interest.

## Supporting information


**Table S1:** Key variables for factor analyses (*N* = 430).
**Table S2:** Key variables for structural equation model, age breakdown and race–ethnicity (*N* = 430). (a) Key variables.
**Table S3:** Summary of fit statistics for two‐ to seven‐factor models.
**Table S4:** Standardized factor loadings for the six‐factor solution.
**Table S5:**: Two‐factor model.
**Table S6:** Three‐factor model.
**Table S7:** Four‐factor model.
**Table S8:** Five‐factor model.
**Table S9:** Seven‐factor model.
**Table S10:** Model fit indices for the CFA model.
**Table S11:** CFA factor loadings.
**Table S12:** CFA covariances for six‐factor model.
**Table S13:** CFA variances for six‐factor model.
**Table S14:** Confirmatory factor analysis loadings with cluster‐robust standard errors (cluster = participant ID).
**Table S15:** Confirmatory factor analysis loadings with bootstrap uncertainty (2000 draws).
**Table S16:** Structural equation model results (Year 5 → Year 6; MLR + FIML + Yuan–Bentler robust corrections).
**Figure S1:** Timeline of data collection for SEM variables. This figure depicts the timeline of data collection for each variable from the NCANDA study used in constructing the structural equation model, with variables colour‐coded by role in the model.

## Data Availability

The data used in this study are part of the National Consortium on Alcohol and Neurodevelopment in Adolescence (NCANDA) collection and are available through the National Institute of Mental Health Data Archive (NDA). Access to these data requires submission of a data use request and approval through the NDA Data Access Committee.
