# Supplementary material for: Modelling Incentive Salience in Adolescent Substance Use: The Influence of Substance Cues, Alcohol Expectancies and Socio‐Environmental Factors
Source: Addict Biol. 2025 Nov 29;30(12):e70106. doi: 10.1111/adb.70106 (PMC12664821; doi:10.1111/adb.70106)
Supplement: Supplementary file 1 — Table S1: Key variables for factor analyses (N = 430). Table S2: Key variables for structural equation model, age breakdown and race–ethnicity (N = 430). (a) Key variables. Table S3: Summary of fit statistics for two‐ to seven‐factor models. Table S4: Standardized factor loadings for the six‐factor solution. Table S5:: Two‐factor model. Table S6: Three‐factor model. Table S7: Four‐factor model. Table S8: Five‐factor model. Table S9: Seven‐factor model. Table S10: Model fit indices for the CFA model. Table S11: CFA factor loadings. Table S12: CFA covariances for six‐factor model. Table S13: CFA variances for six‐factor model. Table S14: Confirmatory factor analysis loadings with cluster‐robust standard errors (cluster = participant ID). Table S15: Confirmatory factor analysis loadings with bootstrap uncertainty (2000 draws). Table S16: Structural equation model results (Year 5 → Year 6; MLR + FIML + Yuan–Bentler robust corrections). Figure S1: Timeline of data collection for SEM variables. This figure depicts the timeline of data collection for each variable from the NCANDA study used in constructing the structural equation model, with variables colour‐coded by role in the model. [file ADB-30-e70106-s001.docx]

**APPENDIX A.** **SUPPORTING INFORMATION**

**Supplementary Table 1**: Key Variables for Factor Analyses (N=430)

| Variable | Mean | Standard Deviation | Min | Max |
| --- | --- | --- | --- | --- |
| ***Urge Regulation*** |  |  |  |  |
| DTCQ 1.1 | 5.4532 | 1.1683 | 0 | 6 |
| DTCQ 1.2 | 5.7493 | 0.9107 | 0 | 6 |
| DTCQ 1.3 | 5.6726 | 0.9578 | 0 | 6 |
| DTCQ 1.4 | 5.6480 | 1.0247 | 0 | 6 |
| DTCQ 2.5 | 5.5705 | 1.0761 | 0 | 6 |
| DTCQ 2.6 | 5.5970 | 1.0610 | 0 | 6 |
| DTCQ 3.1b | 4.9439 | 1.8220 | 0 | 6 |
| DTCQ 3.2b | 4.5598 | 2.0357 | 0 | 6 |
| DTCQ 3.3b | 5.3018 | 1.5451 | 0 | 6 |
| DTCQ 3.4b | 5.2384 | 1.6481 | 0 | 6 |
| DTCQ 4.5b | 4.8610 | 1.8571 | 0 | 6 |
| DTCQ 4.6b | 5.1920 | 1.6371 | 0 | 6 |
| ***Motivational Attitudes*** |  |  |  |  |
| MAAQ 1.1 | 2.8579 | 1.2153 | 0 | 4 |
| MAAQ 1.2 | 2.1143 | 1.4684 | 0 | 4 |
| MAAQ 1.3 | 1.5712 | 1.2833 | 0 | 4 |
| MAAQ 1.4 | 0.7579 | 1.2332 | 0 | 4 |
| ***Expectancies*** |  |  |  |  |
| AEQ 1.1 | 3.1490 | 1.2244 | 1 | 5 |
| AEQ 1.2 | 2.8080 | 1.1220 | 1 | 5 |
| AEQ 1.3 | 2.7234 | 1.1731 | 1 | 5 |
| AEQ 2.10 | 2.7715 | 1.1667 | 1 | 5 |
| AEQ 2.11 | 4.0777 | 1.0070 | 1 | 5 |
| AEQ 2.12 | 3.4910 | 1.0861 | 1 | 5 |
| AEQ 2.13 | 3.9856 | 0.9246 | 1 | 5 |
| AEQ 2.14 | 1.6903 | 0.8447 | 1 | 5 |
| AEQ 3.15 | 3.4775 | 1.2561 | 1 | 5 |
| AEQ 3.16 | 4.1150 | 0.9049 | 1 | 5 |
| AEQ 3.17 | 3.4058 | 1.0323 | 1 | 5 |

This table summarizes the descriptive statistics for variables used in the factor analyses. Variables represent constructs of urge regulation (Drug Taking Confidence Questionnaire, DTCQ), motivational attitudes (Motives for Abstaining from Alcohol Questionnaire, MAAQ), and alcohol expectancies (Alcohol Expectancy Questionnaire, AEQ). These constructs informed the exploratory and confirmatory factor analyses, identifying latent structures related to substance use behaviors.

**Supplementary Table 2**: Key Variables for Structural Equation Model, Age Breakdown, and Race-Ethnicity (N=430)

(a) Key Variables

| Variable | Mean | Standard Deviation | Min | Max |
| --- | --- | --- | --- | --- |
| ***Urge Regulation*** |  |  |  |  |
| DTCQ 1.1 | 5.58 | 1.19 | 0 | 6 |
| DTCQ 1.2 | 5.73 | 1.08 | 0 | 6 |
| DTCQ 1.3 | 5.70 | 1.07 | 0 | 6 |
| DTCQ 1.4 | 5.64 | 1.17 | 0 | 6 |
| DTCQ 2.5 | 5.66 | 1.15 | 0 | 6 |
| DTCQ 2.6 | 5.68 | 1.13 | 0 | 6 |
| DTCQ 3.1b | 5.04 | 1.73 | 0 | 6 |
| DTCQ 3.2b | 4.70 | 1.94 | 0 | 6 |
| DTCQ 3.3b | 5.34 | 1.49 | 0 | 6 |
| DTCQ 3.4b | 5.31 | 1.55 | 0 | 6 |
| DTCQ 4.5b | 4.88 | 1.81 | 0 | 6 |
| DTCQ 4.6b | 5.25 | 1.57 | 0 | 6 |
| ***Motivational Attitudes*** |  |  |  |  |
| MAAQ 1.1 | 2.95 | 1.26 | 0 | 4 |
| MAAQ 1.2 | 2.17 | 1.53 | 0 | 4 |
| MAAQ 1.3 | 1.87 | 1.44 | 0 | 4 |
| MAAQ 1.4 | 1.12 | 1.49 | 0 | 4 |
| ***Expectancies*** |  |  |  |  |
| AEQ 1.1 | 2.87 | 1.27 | 1 | 5 |
| AEQ 1.2 | 2.58 | 1.16 | 1 | 5 |
| AEQ 1.3 | 2.51 | 1.18 | 1 | 5 |
| AEQ 2.10 | 2.62 | 1.21 | 1 | 5 |
| AEQ 2.11 | 3.99 | 1.11 | 1 | 5 |
| AEQ 2.12 | 3.32 | 1.16 | 1 | 5 |
| AEQ 2.13 | 3.86 | 1.05 | 1 | 5 |
| AEQ 2.14 | 1.67 | 0.88 | 1 | 5 |
| AEQ 3.15 | 2.91 | 1.36 | 1 | 5 |
| AEQ 3.16 | 4.01 | 1.05 | 1 | 5 |
| AEQ 3.17 | 3.26 | 1.12 | 1 | 5 |
| ***Outcomes*** |  |  |  |  |
| Onset of Regular Drinking | 0.27 | 0.44 | 0 | 1 |
| Total Number of Drugs Used | 0.33 | 1.05 | 0 | 12 |
| Ever had sexual intercourse? | 0.53 | 0.50 | 0 | 1 |
| Past Year Binge Drinking | 17.88 | 28.17 | 0 | 207 |
| ***Demographics*** |  |  |  |  |
| Visit Age | 19.00 | 3.23 | 12 | 28.18 |
| Total Household Income | 7.68 | 2.66 | 1 | 11 |
| Female | 50.61% |  |  |  |

(b) Age Breakdown

| Variable | Mean | Standard Deviation | Min | Max |
| --- | --- | --- | --- | --- |
| Age of Onset of Regular Drinking | 19.13 | 1.83 | 13.5 | 26 |
| Age of First Sexual Intercourse | 17.07 | 1.96 | 9 | 24 |

(c) Race-Ethnicity

| **Race-Ethnicity** | **Percentage** |
| --- | --- |
| Native American/American Indian | 0.16 |
| Asian | 6.13 |
| Pacific Islander | 0.89 |
| African-American/Black | 8.79 |
| Caucasian/White | 78.63 |
| Other | 5.4 |
| Hispanic | 10.08 |

This table provides descriptive statistics for variables representing latent constructs of urge regulation, motivational attitudes, and alcohol expectancies, as derived from the Drug Taking Confidence Questionnaire (DTCQ), the Motives for Abstaining from Alcohol Questionnaire (MAAQ), and the Alcohol Expectancy Questionnaire (AEQ). These measures form the basis for exploratory and confirmatory factor analyses in this study, which investigates the relationships between incentive salience constructs and substance use behaviors in a sample of youth and young adults. Additionally, the table includes an age breakdown for Age of Onset of Regular Drinking and Age of First Sexual Intercourse. Finally, the table details the racial and ethnic composition of the longitudinal sample.

**Supplementary Table 3:** Summary of Fit Statistics for 2- to 7-Factor Models

| **Number of Factors** | **BIC** | **Degrees of Freedom (df)** | **p-value** | **TLI** | **RMSEA (90% CI)** |
| --- | --- | --- | --- | --- | --- |
| 2 | 238,378.6 | 778 | 0 | 0.514 | 0.121 (0.121–0.121) |
| 3 | 145,338.6 | 738 | 0 | 0.683 | 0.098 (0.097–0.098) |
| 4 | 112,239.4 | 699 | 0 | 0.739 | 0.089 (0.088–0.089) |
| 5 | 91,517.12 | 661 | 0 | 0.773 | 0.083 (0.082–0.083) |
| 6 | 59,095.58 | 624 | 0 | 0.840 | 0.069 (0.069–0.070) |
| 7 | 50,563.08 | 588 | 0 | 0.854 | 0.066 (0.066–0.067) |

This table presents the fit statistics for exploratory factor analyses (EFA) assessing models with 2 to 7 latent factors. Metrics include the Bayesian Information Criterion (BIC), degrees of freedom (df), p-value, Tucker-Lewis Index (TLI), and Root Mean Square Error of Approximation (RMSEA) with 90% confidence intervals (CI). Lower BIC values and RMSEA values closer to 0 indicate better model fit, while higher TLI values suggest improved goodness-of-fit. The 6-factor and 7-factor models show the best fit, as evidenced by the lowest BIC values, acceptable RMSEA (< 0.08), and TLI approaching 0.85. These results informed the selection of the optimal factor structure for subsequent analyses.

**Supplementary Table 4:** Standardized Factor Loadings for the 6-Factor Solution

| **Item** | **Factor 1** | **Factor 2** | **Factor 3** | **Factor 4** | **Factor 5** | **Factor 6** | **Communalities** | **Unique Variance** |
| --- | --- | --- | --- | --- | --- | --- | --- | --- |
| **AEQ.1.1** | 0.03 | **0.71** | 0.00 | 0.04 | -0.06 | -0.07 | **0.51** | 0.49 |
| **AEQ.1.2** | 0.01 | **0.70** | 0.01 | 0.15 | -0.04 | -0.04 | **0.52** | 0.48 |
| **AEQ.1.3** | 0.05 | **0.72** | -0.02 | 0.19 | -0.06 | -0.05 | **0.57** | 0.43 |
| **AEQ.1.4** | 0.04 | **0.58** | -0.02 | **0.39** | -0.07 | -0.04 | **0.49** | 0.51 |
| **AEQ.1.5** | -0.05 | **0.43** | -0.03 | **0.58** | 0.03 | -0.01 | **0.53** | 0.47 |
| **AEQ.1.6** | 0.04 | **0.63** | 0.00 | 0.12 | -0.07 | -0.04 | **0.42** | 0.58 |
| **AEQ.1.7** | -0.02 | **0.60** | 0.01 | -0.01 | -0.01 | 0.01 | **0.36** | 0.64 |
| **AEQ.2.10** | 0.00 | **0.66** | 0.00 | 0.09 | -0.04 | -0.02 | **0.44** | 0.56 |
| **AEQ.2.11** | 0.03 | 0.27 | 0.01 | -0.36 | 0.05 | -0.03 | 0.21 | **0.79** |
| **AEQ.2.12** | 0.12 | **0.68** | 0.01 | -0.22 | -0.07 | 0.02 | **0.53** | 0.47 |
| **AEQ.2.13** | 0.05 | **0.50** | 0.06 | -0.45 | 0.02 | -0.03 | **0.46** | 0.54 |
| **AEQ.2.14** | -0.07 | 0.28 | 0.00 | **0.54** | 0.03 | 0.02 | **0.37** | 0.63 |
| **AEQ.2.8** | -0.01 | **0.62** | 0.03 | 0.08 | 0.02 | 0.00 | **0.40** | 0.60 |
| **AEQ.2.9** | -0.01 | **0.38** | -0.01 | **0.62** | 0.04 | 0.02 | **0.53** | 0.47 |
| **AEQ.3.15** | 0.01 | **0.65** | -0.05 | -0.10 | -0.12 | -0.06 | **0.46** | 0.54 |
| **AEQ.3.16** | 0.03 | **0.30** | 0.04 | -0.50 | 0.02 | -0.04 | **0.34** | 0.66 |
| **AEQ.3.17** | 0.12 | **0.64** | 0.01 | -0.17 | -0.09 | 0.07 | **0.47** | 0.53 |
| **AEQ.3.18** | -0.05 | **0.46** | -0.02 | **0.30** | 0.09 | 0.03 | **0.31** | 0.69 |
| **AEQ.3.19** | -0.02 | **0.60** | -0.02 | -0.01 | -0.03 | 0.03 | **0.36** | 0.64 |
| **AEQ.3.20** | 0.00 | **0.67** | 0.00 | 0.02 | -0.02 | 0.01 | **0.45** | 0.55 |
| **AEQ.3.21** | -0.01 | **0.72** | -0.02 | -0.09 | -0.07 | 0.00 | **0.53** | 0.47 |
| **DTCQ.1.1** | 0.24 | -0.04 | **0.78** | 0.00 | 0.05 | 0.21 | **0.71** | 0.29 |
| **DTCQ.1.2** | 0.18 | 0.03 | **0.80** | -0.05 | 0.02 | -0.05 | **0.68** | 0.32 |
| **DTCQ.1.3** | 0.21 | 0.00 | **0.80** | -0.02 | 0.01 | 0.02 | **0.69** | 0.31 |
| **DTCQ.1.4** | 0.16 | -0.01 | **0.77** | -0.05 | -0.01 | 0.03 | **0.62** | 0.38 |
| **DTCQ.2.5** | 0.17 | 0.00 | **0.80** | 0.00 | 0.02 | 0.20 | **0.72** | 0.28 |
| **DTCQ.2.6** | 0.20 | 0.02 | **0.82** | 0.00 | 0.03 | 0.13 | **0.73** | 0.27 |
| **DTCQ.2.7** | 0.21 | -0.05 | **0.48** | 0.08 | 0.01 | **0.72** | **0.81** | 0.19 |
| **DTCQ.2.8** | 0.23 | -0.07 | **0.43** | 0.07 | -0.01 | **0.73** | **0.78** | 0.22 |
| **DTCQ.3.1b** | **0.89** | 0.04 | 0.18 | -0.05 | 0.00 | -0.01 | **0.83** | 0.17 |
| **DTCQ.3.2b** | **0.80** | 0.04 | 0.13 | -0.01 | 0.00 | 0.08 | **0.66** | 0.34 |
| **DTCQ.3.3b** | **0.80** | 0.04 | 0.28 | -0.06 | 0.00 | -0.11 | **0.73** | 0.27 |
| **DTCQ.3.4b** | **0.80** | 0.05 | 0.21 | -0.05 | 0.01 | -0.09 | **0.69** | 0.31 |
| **DTCQ.4.5b** | **0.81** | 0.01 | 0.23 | -0.04 | 0.01 | 0.12 | **0.73** | 0.27 |
| **DTCQ.4.6b** | **0.85** | 0.03 | 0.25 | -0.04 | 0.01 | -0.09 | **0.79** | 0.21 |
| **DTCQ.4.7b** | **0.77** | 0.01 | 0.06 | 0.01 | -0.02 | **0.38** | **0.75** | 0.25 |
| **DTCQ.4.8b** | **0.78** | 0.02 | 0.06 | -0.01 | -0.03 | **0.36** | **0.75** | 0.25 |
| **MAAQ.1.1** | 0.05 | -0.06 | 0.02 | -0.10 | **0.54** | -0.06 | **0.31** | 0.69 |
| **MAAQ.1.2** | 0.01 | 0.01 | 0.00 | 0.04 | **0.70** | -0.01 | **0.49** | 0.51 |
| **MAAQ.1.3** | 0.00 | -0.01 | -0.02 | 0.05 | **0.72** | -0.05 | **0.53** | 0.47 |
| **MAAQ.1.4** | -0.01 | -0.07 | -0.02 | 0.06 | **0.56** | 0.04 | **0.32** | 0.68 |
| **MAAQ.1.5** | -0.06 | -0.16 | 0.08 | -0.05 | **0.43** | 0.06 | **0.22** | 0.78 |

This table summarizes the standardized factor loadings for a 6-factor solution identified through confirmatory factor analysis. Each item is associated with its respective latent factor (Factor 1 to Factor 6), which reflect dimensions of incentive salience constructs such as alcohol expectancies, drug urge regulation, and motivational attitudes. The table also includes communalities, representing the proportion of variance explained by the factors, and unique variance, indicating the variance not captured by the factors. Strong loadings (e.g., > 0.50) highlight items that strongly contribute to each factor. These findings validate the robust structure of latent constructs used to model substance use behaviors and related outcomes.

*DTCQ: Drug Taking Confidence Questionnaire; MAAQ: Motives for Abstaining from Alcohol Questionnaire; AEQ: Alcohol Expectancy Questionnaire.*

**Supplementary Table 5**: 2-Factor Model

| Variable | Factor 1 | Factor 2 | Unique Variance | Communalities |
| --- | --- | --- | --- | --- |
| AEQ 1.1 | 0.01 | 0.72 | 0.49 | 0.51 |
| AEQ 1.2 | 0 | 0.72 | 0.48 | 0.52 |
| AEQ 1.3 | 0.01 | 0.75 | 0.44 | 0.56 |
| AEQ 1.4 | -0.01 | 0.61 | 0.62 | 0.38 |
| AEQ 1.5 | -0.08 | 0.47 | 0.77 | 0.23 |
| AEQ 1.6 | 0.02 | 0.65 | 0.58 | 0.42 |
| AEQ 1.7 | -0.01 | 0.59 | 0.65 | 0.35 |
| AEQ 2.10 | -0.01 | 0.67 | 0.56 | 0.44 |
| AEQ 2.11 | 0.05 | 0.22 | 0.95 | 0.05 |
| AEQ 2.12 | 0.12 | 0.65 | 0.56 | 0.44 |
| AEQ 2.13 | 0.1 | 0.43 | 0.8 | 0.2 |
| AEQ 2.14 | -0.08 | 0.32 | 0.89 | 0.11 |
| AEQ 2.8 | 0.01 | 0.62 | 0.61 | 0.39 |
| AEQ 2.9 | -0.05 | 0.41 | 0.83 | 0.17 |
| AEQ 3.15 | -0.03 | 0.65 | 0.57 | 0.43 |
| AEQ 3.16 | 0.06 | 0.23 | 0.94 | 0.06 |
| AEQ 3.17 | 0.13 | 0.62 | 0.59 | 0.41 |
| AEQ 3.18 | -0.06 | 0.47 | 0.78 | 0.22 |
| AEQ 3.19 | -0.01 | 0.59 | 0.65 | 0.35 |
| AEQ 3.20 | 0 | 0.67 | 0.55 | 0.45 |
| AEQ 3.21 | -0.01 | 0.71 | 0.5 | 0.5 |
| DTCQ 1.1 | 0.69 | -0.08 | 0.52 | 0.48 |
| DTCQ 1.2 | 0.6 | 0 | 0.64 | 0.36 |
| DTCQ 1.3 | 0.64 | -0.03 | 0.59 | 0.41 |
| DTCQ 1.4 | 0.58 | -0.04 | 0.66 | 0.34 |
| DTCQ 2.5 | 0.65 | -0.04 | 0.58 | 0.42 |
| DTCQ 2.6 | 0.67 | -0.01 | 0.56 | 0.44 |
| DTCQ 2.7 | 0.58 | -0.08 | 0.66 | 0.34 |
| DTCQ 2.8 | 0.56 | -0.09 | 0.68 | 0.32 |
| DTCQ 3.1b | 0.78 | 0.07 | 0.39 | 0.61 |
| DTCQ 3.2b | 0.7 | 0.07 | 0.51 | 0.49 |
| DTCQ 3.3b | 0.76 | 0.06 | 0.42 | 0.58 |
| DTCQ 3.4b | 0.71 | 0.07 | 0.48 | 0.52 |
| DTCQ 4.5b | 0.79 | 0.03 | 0.38 | 0.62 |
| DTCQ 4.6b | 0.78 | 0.06 | 0.39 | 0.61 |
| DTCQ 4.7b | 0.7 | 0.03 | 0.51 | 0.49 |
| DTCQ 4.8b | 0.7 | 0.04 | 0.5 | 0.5 |
| MAAQ 1.1 | 0.05 | -0.11 | 0.99 | 0.01 |
| MAAQ 1.2 | 0.01 | -0.05 | 1 | 0 |
| MAAQ 1.3 | -0.02 | -0.07 | 1 | 0 |
| MAAQ 1.4 | -0.01 | -0.11 | 0.99 | 0.01 |
| MAAQ 1.5 | 0.02 | -0.21 | 0.95 | 0.05 |

**Supplementary Table 6:** 3-Factor Model

| Variable | Factor 1 | Factor 2 | Factor 3 | Unique Variance | Communalities |
| --- | --- | --- | --- | --- | --- |
| MAAQ1.1 | 0.03 | 0.71 | -0.02 | 0.49 | 0.5113 |
| MAAQ1.2 | -0.01 | 0.72 | 0 | 0.48 | 0.5219 |
| MAAQ1.3 | 0.03 | 0.75 | -0.03 | 0.44 | 0.5584 |
| MAAQ1.4 | -0.01 | 0.62 | -0.01 | 0.62 | 0.3797 |
| MAAQ1.5 | -0.12 | 0.48 | 0.01 | 0.76 | 0.2414 |
| AEQ1.1 | 0.03 | 0.65 | 0 | 0.58 | 0.424 |
| AEQ1.2 | -0.01 | 0.59 | 0.01 | 0.65 | 0.3508 |
| AEQ1.3 | 0.03 | 0.67 | 0 | 0.55 | 0.4451 |
| AEQ2.10 | -0.01 | 0.67 | 0 | 0.55 | 0.4451 |
| AEQ2.11 | 0.08 | 0.22 | -0.02 | 0.95 | 0.0542 |
| AEQ2.12 | 0.16 | 0.64 | -0.01 | 0.56 | 0.4423 |
| AEQ2.13 | 0.11 | 0.43 | 0.02 | 0.8 | 0.196 |
| AEQ2.14 | -0.14 | 0.33 | 0.04 | 0.87 | 0.1271 |
| AEQ3.15 | 0.03 | 0.65 | -0.08 | 0.57 | 0.4278 |
| AEQ3.16 | 0.09 | 0.23 | -0.01 | 0.94 | 0.0604 |
| AEQ3.17 | 0.16 | 0.62 | 0.01 | 0.59 | 0.4086 |
| AEQ3.18 | -0.09 | 0.47 | 0.01 | 0.77 | 0.2331 |
| AEQ3.19 | -0.01 | 0.59 | -0.01 | 0.65 | 0.3485 |
| AEQ3.20 | 0 | 0.67 | 0 | 0.55 | 0.4492 |
| AEQ3.21 | 0.01 | 0.71 | -0.03 | 0.5 | 0.4994 |
| DTCQ1.1 | 0.22 | -0.05 | 0.82 | 0.28 | 0.7218 |
| DTCQ1.2 | 0.17 | 0.03 | 0.75 | 0.41 | 0.5882 |
| DTCQ1.3 | 0.19 | 0 | 0.78 | 0.36 | 0.6434 |
| DTCQ1.4 | 0.15 | -0.01 | 0.74 | 0.43 | 0.5665 |
| DTCQ2.5 | 0.16 | 0 | 0.84 | 0.27 | 0.7281 |
| DTCQ2.6 | 0.19 | 0.02 | 0.83 | 0.27 | 0.7298 |
| DTCQ2.7 | 0.23 | -0.06 | 0.63 | 0.56 | 0.4446 |
| DTCQ2.8 | 0.24 | -0.08 | 0.57 | 0.6 | 0.3959 |
| DTCQ3.1b | 0.88 | 0.04 | 0.18 | 0.19 | 0.8145 |
| DTCQ3.2b | 0.79 | 0.04 | 0.16 | 0.34 | 0.6576 |
| DTCQ3.3b | 0.78 | 0.04 | 0.25 | 0.32 | 0.6787 |
| DTCQ3.4b | 0.78 | 0.05 | 0.19 | 0.35 | 0.653 |
| DTCQ4.5b | 0.81 | 0.01 | 0.27 | 0.27 | 0.7326 |
| DTCQ4.6b | 0.83 | 0.04 | 0.24 | 0.26 | 0.743 |
| DTCQ4.7b | 0.76 | 0 | 0.19 | 0.38 | 0.616 |
| DTCQ4.8b | 0.78 | 0.02 | 0.18 | 0.36 | 0.6391 |

**Supplementary Table 7**: 4-Factor Model

| Variable | Factor 1 | Factor 2 | Factor 3 | Factor 4 | Unique Variance | Communalities |
| --- | --- | --- | --- | --- | --- | --- |
| AEQ1.1 | 0.69 | 0.03 | -0.01 | 0.19 | 0.49 | 0.509 |
| AEQ1.2 | 0.72 | 0.01 | 0.00 | 0.07 | 0.48 | 0.522 |
| AEQ1.3 | 0.75 | 0.05 | -0.03 | 0.05 | 0.43 | 0.567 |
| AEQ1.4 | 0.67 | 0.04 | -0.03 | -0.18 | 0.52 | 0.480 |
| AEQ1.5 | 0.59 | -0.04 | -0.03 | -0.42 | 0.47 | 0.526 |
| AEQ1.6 | 0.64 | 0.04 | 0.00 | 0.09 | 0.58 | 0.422 |
| AEQ1.7 | 0.57 | -0.02 | 0.02 | 0.18 | 0.65 | 0.354 |
| AEQ2.10 | 0.65 | 0.00 | 0.00 | 0.11 | 0.56 | 0.442 |
| AEQ2.11 | 0.15 | 0.02 | 0.02 | 0.41 | 0.81 | 0.187 |
| AEQ2.12 | 0.58 | 0.12 | 0.02 | 0.41 | 0.48 | 0.519 |
| AEQ2.13 | 0.34 | 0.04 | 0.06 | 0.55 | 0.58 | 0.424 |
| AEQ2.14 | 0.43 | -0.06 | 0.00 | -0.43 | 0.63 | 0.373 |
| AEQ2.8 | 0.62 | -0.01 | 0.04 | 0.10 | 0.61 | 0.390 |
| AEQ2.9 | 0.54 | 0.00 | -0.01 | -0.48 | 0.47 | 0.529 |
| AEQ3.15 | 0.60 | 0.00 | -0.06 | 0.31 | 0.54 | 0.461 |
| AEQ3.16 | 0.13 | 0.02 | 0.04 | 0.55 | 0.68 | 0.316 |
| AEQ3.17 | 0.56 | 0.13 | 0.03 | 0.35 | 0.54 | 0.457 |
| AEQ3.18 | 0.52 | -0.04 | -0.01 | -0.18 | 0.70 | 0.305 |
| AEQ3.19 | 0.56 | -0.01 | 0.00 | 0.18 | 0.65 | 0.351 |
| AEQ3.20 | 0.65 | 0.00 | 0.01 | 0.17 | 0.55 | 0.448 |
| AEQ3.21 | 0.66 | -0.01 | -0.01 | 0.29 | 0.47 | 0.525 |
| DTCQ1.1 | -0.05 | 0.23 | 0.81 | -0.03 | 0.28 | 0.720 |
| DTCQ1.2 | 0.01 | 0.16 | 0.76 | 0.09 | 0.39 | 0.610 |
| DTCQ1.3 | -0.01 | 0.19 | 0.78 | 0.04 | 0.35 | 0.653 |
| DTCQ1.4 | -0.03 | 0.14 | 0.75 | 0.07 | 0.41 | 0.587 |
| DTCQ2.5 | -0.01 | 0.17 | 0.84 | -0.01 | 0.27 | 0.729 |
| DTCQ2.6 | 0.01 | 0.19 | 0.83 | 0.01 | 0.27 | 0.732 |
| DTCQ2.7 | -0.03 | 0.26 | 0.61 | -0.18 | 0.53 | 0.469 |
| DTCQ2.8 | -0.05 | 0.28 | 0.56 | -0.18 | 0.58 | 0.421 |
| DTCQ3.1b | 0.02 | 0.88 | 0.17 | 0.09 | 0.19 | 0.815 |
| DTCQ3.2b | 0.03 | 0.80 | 0.15 | 0.03 | 0.33 | 0.667 |
| DTCQ3.3b | 0.01 | 0.77 | 0.25 | 0.12 | 0.32 | 0.676 |
| DTCQ3.4b | 0.02 | 0.78 | 0.19 | 0.10 | 0.35 | 0.651 |
| DTCQ4.5b | -0.01 | 0.82 | 0.26 | 0.04 | 0.26 | 0.738 |
| DTCQ4.6b | 0.01 | 0.82 | 0.23 | 0.09 | 0.26 | 0.741 |
| DTCQ4.7b | 0.01 | 0.79 | 0.16 | -0.05 | 0.35 | 0.651 |
| DTCQ4.8b | 0.01 | 0.80 | 0.16 | -0.02 | 0.34 | 0.664 |
| MAAQ1.1 | -0.11 | 0.03 | 0.03 | -0.02 | 0.99 | 0.014 |
| MAAQ1.2 | -0.02 | 0.00 | 0.03 | -0.14 | 0.98 | 0.022 |
| MAAQ1.3 | -0.04 | -0.02 | 0.01 | -0.15 | 0.97 | 0.025 |
| MAAQ1.4 | -0.08 | -0.02 | 0.02 | -0.18 | 0.96 | 0.038 |
| MAAQ1.5 | -0.19 | -0.06 | 0.11 | -0.09 | 0.94 | 0.060 |

**Supplementary Table 8**: 5-Factor Model

| Variable | Factor 1 | Factor 2 | Factor 3 | Factor 4 | Factor 5 | Unique Variance | Communalities |
| --- | --- | --- | --- | --- | --- | --- | --- |
| AEQ1.1 | 0.71 | 0.03 | -0.02 | 0.00 | -0.05 | 0.49 | 0.51 |
| AEQ1.2 | 0.71 | 0.01 | -0.01 | 0.12 | -0.04 | 0.48 | 0.52 |
| AEQ1.3 | 0.73 | 0.05 | -0.04 | 0.16 | -0.06 | 0.43 | 0.57 |
| AEQ1.4 | 0.59 | 0.03 | -0.03 | 0.36 | -0.06 | 0.52 | 0.48 |
| AEQ1.5 | 0.46 | -0.05 | -0.02 | 0.56 | 0.03 | 0.48 | 0.52 |
| AEQ1.6 | 0.64 | 0.03 | -0.01 | 0.09 | -0.06 | 0.58 | 0.42 |
| AEQ1.7 | 0.60 | -0.02 | 0.01 | -0.03 | -0.02 | 0.64 | 0.36 |
| AEQ2.10 | 0.66 | 0.00 | 0.00 | 0.06 | -0.04 | 0.56 | 0.44 |
| AEQ2.11 | 0.26 | 0.03 | 0.00 | -0.37 | 0.04 | 0.79 | 0.21 |
| AEQ2.12 | 0.67 | 0.13 | 0.01 | -0.23 | -0.07 | 0.48 | 0.52 |
| AEQ2.13 | 0.48 | 0.05 | 0.04 | -0.46 | 0.02 | 0.55 | 0.45 |
| AEQ2.14 | 0.30 | -0.07 | 0.01 | 0.52 | 0.04 | 0.63 | 0.37 |
| AEQ2.8 | 0.63 | -0.01 | 0.03 | 0.05 | 0.02 | 0.60 | 0.40 |
| AEQ2.9 | 0.40 | -0.02 | 0.00 | 0.60 | 0.04 | 0.48 | 0.52 |
| AEQ3.15 | 0.65 | 0.00 | -0.07 | -0.12 | -0.12 | 0.54 | 0.46 |
| AEQ3.16 | 0.28 | 0.03 | 0.02 | -0.51 | 0.02 | 0.66 | 0.34 |
| AEQ3.17 | 0.63 | 0.13 | 0.02 | -0.18 | -0.10 | 0.54 | 0.46 |
| AEQ3.18 | 0.47 | -0.05 | -0.01 | 0.29 | 0.09 | 0.69 | 0.31 |
| AEQ3.19 | 0.59 | -0.01 | -0.01 | -0.02 | -0.03 | 0.65 | 0.35 |
| AEQ3.20 | 0.67 | 0.00 | 0.00 | 0.00 | -0.02 | 0.55 | 0.45 |
| AEQ3.21 | 0.71 | -0.01 | -0.02 | -0.10 | -0.07 | 0.48 | 0.52 |
| DTCQ1.1 | -0.04 | 0.23 | 0.82 | -0.01 | 0.05 | 0.28 | 0.72 |
| DTCQ1.2 | 0.04 | 0.16 | 0.76 | -0.11 | 0.04 | 0.39 | 0.61 |
| DTCQ1.3 | 0.01 | 0.19 | 0.78 | -0.07 | 0.03 | 0.35 | 0.65 |
| DTCQ1.4 | 0.00 | 0.14 | 0.75 | -0.10 | 0.01 | 0.41 | 0.59 |
| DTCQ2.5 | 0.00 | 0.16 | 0.84 | -0.01 | 0.02 | 0.27 | 0.73 |
| DTCQ2.6 | 0.03 | 0.19 | 0.83 | -0.03 | 0.04 | 0.27 | 0.73 |
| DTCQ2.7 | -0.07 | 0.25 | 0.62 | 0.16 | -0.03 | 0.52 | 0.48 |
| DTCQ2.8 | -0.09 | 0.27 | 0.57 | 0.16 | -0.04 | 0.57 | 0.43 |
| DTCQ3.1b | 0.04 | 0.88 | 0.17 | -0.07 | 0.01 | 0.18 | 0.82 |
| DTCQ3.2b | 0.04 | 0.80 | 0.15 | -0.01 | 0.00 | 0.33 | 0.67 |
| DTCQ3.3b | 0.05 | 0.78 | 0.25 | -0.11 | 0.02 | 0.32 | 0.68 |
| DTCQ3.4b | 0.06 | 0.78 | 0.18 | -0.09 | 0.02 | 0.35 | 0.65 |
| DTCQ4.5b | 0.01 | 0.82 | 0.26 | -0.03 | 0.00 | 0.26 | 0.74 |
| DTCQ4.6b | 0.04 | 0.83 | 0.23 | -0.08 | 0.03 | 0.26 | 0.74 |
| DTCQ4.7b | -0.01 | 0.79 | 0.17 | 0.07 | -0.04 | 0.35 | 0.65 |
| DTCQ4.8b | 0.00 | 0.80 | 0.17 | 0.05 | -0.05 | 0.33 | 0.67 |
| MAAQ1.1 | -0.06 | 0.05 | 0.00 | -0.11 | 0.54 | 0.69 | 0.31 |
| MAAQ1.2 | 0.01 | 0.01 | 0.00 | 0.04 | 0.69 | 0.52 | 0.48 |
| MAAQ1.3 | -0.01 | 0.00 | -0.03 | 0.04 | 0.73 | 0.47 | 0.53 |
| MAAQ1.4 | -0.07 | -0.01 | 0.00 | 0.07 | 0.55 | 0.69 | 0.31 |
| MAAQ1.5 | -0.17 | -0.06 | 0.10 | -0.03 | 0.42 | 0.78 | 0.22 |

**Supplementary Table 9**: 7-Factor Model

| Variable | Factor 1 | Factor 2 | Factor 3 | Factor 4 | Factor 5 | Factor 6 | Factor 7 | Unique Variance | Communalities |
| --- | --- | --- | --- | --- | --- | --- | --- | --- | --- |
| AEQ.1.1 | 0.03 | 0.76 | 0.01 | 0.07 | -0.04 | -0.05 | -0.02 | 0.41 | 0.59 |
| AEQ.1.2 | 0.01 | 0.76 | 0.01 | 0 | -0.02 | -0.02 | 0.08 | 0.42 | 0.58 |
| AEQ.1.3 | 0.05 | 0.78 | -0.02 | -0.02 | -0.05 | -0.03 | 0.11 | 0.37 | 0.63 |
| AEQ.1.4 | 0.04 | 0.63 | -0.02 | -0.15 | -0.06 | -0.02 | 0.29 | 0.5 | 0.5 |
| AEQ.1.5 | -0.04 | 0.46 | -0.03 | -0.24 | 0.02 | -0.01 | 0.5 | 0.48 | 0.52 |
| AEQ.1.6 | 0.04 | 0.66 | 0 | 0.04 | -0.06 | -0.03 | 0.09 | 0.55 | 0.45 |
| AEQ.1.7 | -0.02 | 0.59 | 0.01 | 0.16 | -0.01 | 0.02 | 0.03 | 0.63 | 0.37 |
| AEQ.2.10 | 0 | 0.62 | 0 | 0.15 | -0.04 | -0.01 | 0.16 | 0.56 | 0.44 |
| AEQ.2.11 | 0.03 | 0.14 | 0.01 | 0.45 | 0.03 | -0.04 | -0.09 | 0.77 | 0.23 |
| AEQ.2.12 | 0.13 | 0.53 | 0 | 0.49 | -0.09 | 0.02 | 0.08 | 0.45 | 0.55 |
| AEQ.2.13 | 0.05 | 0.33 | 0.06 | 0.62 | 0 | -0.04 | -0.08 | 0.5 | 0.5 |
| AEQ.2.14 | -0.06 | 0.22 | 0 | -0.13 | 0 | 0.01 | 0.64 | 0.52 | 0.48 |
| AEQ.2.8 | -0.01 | 0.57 | 0.03 | 0.18 | 0.01 | 0.01 | 0.19 | 0.61 | 0.39 |
| AEQ.2.9 | -0.01 | 0.36 | -0.01 | -0.21 | 0.02 | 0.01 | 0.62 | 0.43 | 0.57 |
| AEQ.3.15 | 0 | 0.65 | -0.05 | 0.2 | -0.11 | -0.04 | -0.06 | 0.51 | 0.49 |
| AEQ.3.16 | 0.03 | 0.19 | 0.03 | 0.49 | 0.02 | -0.04 | -0.25 | 0.66 | 0.34 |
| AEQ.3.17 | 0.13 | 0.51 | 0 | 0.43 | -0.11 | 0.07 | 0.1 | 0.51 | 0.49 |
| AEQ.3.18 | -0.04 | 0.35 | -0.03 | 0.13 | 0.06 | 0.02 | 0.52 | 0.59 | 0.41 |
| AEQ.3.19 | -0.01 | 0.45 | -0.02 | 0.38 | -0.06 | 0.03 | 0.28 | 0.57 | 0.43 |
| AEQ.3.20 | 0 | 0.58 | 0 | 0.28 | -0.03 | 0.02 | 0.21 | 0.54 | 0.46 |
| AEQ.3.21 | -0.01 | 0.63 | -0.02 | 0.33 | -0.08 | 0 | 0.1 | 0.47 | 0.53 |
| DTCQ.1.1 | 0.24 | -0.06 | 0.78 | 0.01 | 0.04 | 0.21 | 0.02 | 0.29 | 0.71 |
| DTCQ.1.2 | 0.18 | 0.02 | 0.8 | 0.05 | 0.02 | -0.05 | -0.02 | 0.32 | 0.68 |
| DTCQ.1.3 | 0.21 | -0.01 | 0.8 | 0.02 | 0.01 | 0.02 | 0 | 0.31 | 0.69 |
| DTCQ.1.4 | 0.16 | -0.01 | 0.77 | 0.02 | 0 | 0.03 | -0.06 | 0.38 | 0.62 |
| DTCQ.2.5 | 0.17 | -0.01 | 0.8 | 0.01 | 0.01 | 0.2 | 0 | 0.28 | 0.72 |
| DTCQ.2.6 | 0.2 | 0.02 | 0.82 | 0.01 | 0.03 | 0.13 | 0 | 0.27 | 0.73 |
| DTCQ.2.7 | 0.21 | -0.04 | 0.49 | -0.07 | 0.01 | 0.72 | 0.04 | 0.19 | 0.81 |
| DTCQ.2.8 | 0.23 | -0.06 | 0.43 | -0.08 | 0 | 0.73 | 0.03 | 0.22 | 0.78 |
| DTCQ.3.1 | 0.89 | 0.03 | 0.17 | 0.05 | 0 | -0.01 | -0.03 | 0.17 | 0.83 |
| DTCQ.3.2 | 0.8 | 0.04 | 0.13 | 0.02 | 0 | 0.08 | -0.01 | 0.34 | 0.66 |
| DTCQ.3.3 | 0.8 | 0.02 | 0.28 | 0.07 | 0 | -0.11 | -0.02 | 0.26 | 0.74 |
| DTCQ.3.4 | 0.8 | 0.04 | 0.21 | 0.05 | 0.01 | -0.09 | -0.03 | 0.31 | 0.69 |
| DTCQ.4.5 | 0.81 | -0.01 | 0.23 | 0.05 | 0 | 0.12 | 0 | 0.27 | 0.73 |
| DTCQ.4.6 | 0.85 | 0.02 | 0.25 | 0.05 | 0.01 | -0.09 | -0.01 | 0.21 | 0.79 |
| DTCQ.4.7 | 0.77 | 0.01 | 0.06 | -0.02 | -0.02 | 0.39 | -0.01 | 0.25 | 0.75 |
| DTCQ.4.8 | 0.78 | 0.03 | 0.06 | -0.01 | -0.03 | 0.37 | -0.05 | 0.24 | 0.76 |
| MAAQ.1.1 | 0.05 | -0.06 | 0.02 | 0.05 | 0.54 | -0.06 | -0.06 | 0.69 | 0.31 |
| MAAQ.1.2 | 0.01 | 0.01 | 0 | -0.02 | 0.7 | 0 | 0.03 | 0.51 | 0.49 |
| MAAQ.1.3 | 0 | 0 | -0.02 | -0.03 | 0.73 | -0.05 | 0.04 | 0.47 | 0.53 |
| MAAQ.1.4 | -0.01 | -0.06 | -0.02 | -0.05 | 0.56 | 0.04 | 0.05 | 0.68 | 0.32 |
| MAAQ.1.5 | -0.06 | -0.19 | 0.08 | 0.03 | 0.42 | 0.06 | 0 | 0.77 | 0.23 |

**Supplementary Table 10:** Model Fit Indices for the CFA Model

| Fit Measure | Value |
| --- | --- |
| Chi-Square (χ²) | 32,705.164 |
| Scaled Chi-Square | 25,347.564 |
| Degrees of Freedom | 310 |
| P-value (Chi-square) | 0.000 |
| Comparative Fit Index (CFI) | 0.906 |
| Robust Comparative Fit Index (CFI) | 0.906 |
| Tucker-Lewis Index (TLI) | 0.893 |
| Robust Tucker-Lewis Index (TLI) | 0.893 |
| Akaike Information Criterion (AIC) | 1,344,644.585 |
| Bayesian Information Criterion (BIC) | 1,345,187.148 |
| Sample-size Adjusted BIC (SABIC) | 1,344,971.047 |
| Root Mean Square Error of Approximation (RMSEA) | 0.070 |
| Robust RMSEA | 0.070 |
| RMSEA 90% CI Lower | 0.069 |
| RMSEA 90% CI Upper | 0.070 |
| P-value H0 (RMSEA ≤ 0.05) | 0.000 |
| P-value H0 (RMSEA ≥ 0.08) | 0.000 |
| Standardized Root Mean Square Residual (SRMR) | 0.051 |

This table presents model fit indices for the confirmatory factor analysis (CFA) used to evaluate the 6-factor solution. Fit measures include Chi-Square (χ²), Comparative Fit Index (CFI), Tucker-Lewis Index (TLI), Akaike Information Criterion (AIC), Bayesian Information Criterion (BIC), and Root Mean Square Error of Approximation (RMSEA) with 90% confidence intervals. Key indices, such as a CFI of 0.906 and RMSEA of 0.070, indicate acceptable fit, while the SRMR value of 0.051 reflects adequate residual variance. These results support the validity of the latent factor structure and its application in modeling incentive salience constructs associated with substance use behaviors.

**Supplementary Table 11:** CFA Factor Loadings

| Latent Variable | Indicator | Estimate | Std. Err | z-value | P(>\|z\|) | Std.lv | Std.all |
| --- | --- | --- | --- | --- | --- | --- | --- |
| Factor 1 | DTCQ 1.1 | 1 |  |  |  | 0.854 | 0.844 |
| Factor 1 | DTCQ 1.2 | 0.99 | 0.015 | 64.825 | 0 | 0.845 | 0.827 |
| Factor 1 | DTCQ 1.3 | 1.007 | 0.013 | 76.431 | 0 | 0.86 | 0.842 |
| Factor 1 | DTCQ 1.4 | 0.951 | 0.014 | 69.615 | 0 | 0.812 | 0.801 |
| Factor 1 | DTCQ 2.5 | 1.018 | 0.009 | 115.583 | 0 | 0.87 | 0.857 |
| Factor 1 | DTCQ 2.6 | 1.036 | 0.008 | 125.112 | 0 | 0.885 | 0.875 |
| Factor 2 | DTCQ 3.1 | 1 |  |  |  | 0.893 | 0.893 |
| Factor 2 | DTCQ 3.2 | 0.86 | 0.005 | 182.686 | 0 | 0.768 | 0.769 |
| Factor 2 | DTCQ 3.3 | 0.978 | 0.008 | 120.183 | 0 | 0.873 | 0.869 |
| Factor 2 | DTCQ 3.4 | 0.971 | 0.008 | 128.594 | 0 | 0.867 | 0.866 |
| Factor 2 | DTCQ 4.5 | 0.926 | 0.006 | 161.193 | 0 | 0.826 | 0.826 |
| Factor 2 | DTCQ 4.6 | 1.027 | 0.006 | 165.132 | 0 | 0.917 | 0.916 |
| Factor 3 | MAAQ 1.1 | 1 |  |  |  | 0.525 | 0.526 |
| Factor 3 | MAAQ 1.2 | 1.367 | 0.019 | 72.024 | 0 | 0.718 | 0.72 |
| Factor 3 | MAAQ 1.3 | 1.399 | 0.028 | 49.68 | 0 | 0.735 | 0.735 |
| Factor 3 | MAAQ 1.4 | 1.034 | 0.025 | 41.243 | 0 | 0.543 | 0.542 |
| Factor 4 | AEQ 1.1 | 1 |  |  |  | 0.748 | 0.749 |
| Factor 4 | AEQ 1.2 | 1.037 | 0.008 | 127.688 | 0 | 0.776 | 0.773 |
| Factor 4 | AEQ 1.3 | 1.05 | 0.009 | 114.498 | 0 | 0.786 | 0.788 |
| Factor 4 | AEQ 2.10 | 0.835 | 0.01 | 80.977 | 0 | 0.625 | 0.627 |
| Factor 5 | AEQ 2.11 | 1 |  |  |  | 0.306 | 0.307 |
| Factor 5 | AEQ 2.12 | 2.784 | 0.105 | 26.403 | 0 | 0.852 | 0.854 |
| Factor 5 | AEQ 2.13 | 1.773 | 0.048 | 36.633 | 0 | 0.543 | 0.548 |
| Factor 5 | AEQ 2.14 | 0.467 | 0.03 | 15.608 | 0 | 0.143 | 0.143 |
| Factor 5 | AEQ 3.15 | 1.85 | 0.072 | 25.835 | 0 | 0.566 | 0.567 |
| Factor 5 | AEQ 3.16 | 1.042 | 0.032 | 32.799 | 0 | 0.319 | 0.322 |
| Factor 5 | AEQ 3.17 | 2.59 | 0.101 | 25.607 | 0 | 0.793 | 0.795 |

This table provides the standardized factor loadings, standard errors, z-values, and p-values for indicators of latent variables identified in the CFA model. Each latent variable (Factor 1 through Factor 5) is defined by multiple indicators derived from the Drug Taking Confidence Questionnaire (DTCQ), Motives for Abstaining from Alcohol Questionnaire (MAAQ), and Alcohol Expectancy Questionnaire (AEQ). The Estimate column represents the unstandardized factor loading, while Std.lv and Std.all columns reflect the standardized loadings on the latent variable and across all indicators, respectively. High standardized loadings (e.g., > 0.50) indicate strong relationships between the indicators and their respective latent factors. These findings support the validity of the CFA model in capturing key constructs related to incentive salience and substance use behaviors.

**Supplementary Table 12**: CFA Covariances for 6-Factor Model

| Variables | Estimate | Std.Err | z-value | P(>\|z\|) | Std.lv | Std.all |
| --- | --- | --- | --- | --- | --- | --- |
| DTCQ 1.1 ~~ DTCQ 1.2 | -0.032 | 0.006 | -5.797 | 0 | -0.032 | -0.103 |
| AEQ 1.1 ~~ AEQ 1.2 | 0.053 | 0.006 | 8.908 | 0 | 0.053 | 0.126 |
| DTCQ 3.1b ~~ DTCQ 3.2 | 0.087 | 0.005 | 18.958 | 0 | 0.087 | 0.302 |
| AEQ 3.15 ~~ AEQ 3.16 | 0.077 | 0.007 | 11.202 | 0 | 0.077 | 0.1 |
| Factor 1 ~~ Factor 2 | 0.348 | 0.011 | 32.262 | 0 | 0.457 | 0.457 |
| Factor 1 ~~ Factor 3 | 0 | 0.004 | -0.02 | 0.984 | 0 | 0 |
| Factor 1 ~~ Factor 4 | -0.004 | 0.005 | -0.743 | 0.457 | -0.006 | -0.006 |
| Factor 1 ~~ Factor 5 | 0.018 | 0.003 | 6.62 | 0 | 0.068 | 0.068 |
| Factor 2 ~~ Factor 3 | -0.009 | 0.004 | -2.26 | 0.024 | -0.019 | -0.019 |
| Factor 2 ~~ Factor 4 | 0.044 | 0.005 | 8.118 | 0 | 0.066 | 0.066 |
| Factor 2 ~~ Factor 5 | 0.046 | 0.003 | 15.541 | 0 | 0.169 | 0.169 |
| Factor 3 ~~ Factor 4 | -0.026 | 0.004 | -7.296 | 0 | -0.067 | -0.067 |
| Factor 3 ~~ Factor 5 | -0.02 | 0.002 | -11.624 | 0 | -0.124 | -0.124 |
| Factor 4 ~~ Factor 5 | 0.163 | 0.007 | 24.39 | 0 | 0.712 | 0.712 |

**Supplementary Table 13**: CFA Variances for 6-Factor Model

| Variable | Estimate | Std.Err | z-value | P(>\|z\|) |
| --- | --- | --- | --- | --- |
| DTCQ 1.1 | 0.295 | 0.009 | 33.514 | 0 |
| DTCQ 1.2 | 0.331 | 0.011 | 30.003 | 0 |
| DTCQ 1.3 | 0.303 | 0.008 | 40.005 | 0 |
| DTCQ 1.4 | 0.37 | 0.011 | 32.18 | 0 |
| DTCQ 2.5 | 0.273 | 0.009 | 31.383 | 0 |
| DTCQ 2.6 | 0.239 | 0.008 | 30.603 | 0 |
| DTCQ 3.1 | 0.203 | 0.006 | 35.268 | 0 |
| DTCQ 3.2 | 0.407 | 0.007 | 56.636 | 0 |
| DTCQ 3.3 | 0.247 | 0.006 | 38.259 | 0 |
| DTCQ 3.4 | 0.251 | 0.007 | 37.957 | 0 |
| DTCQ 4.5 | 0.317 | 0.007 | 47.635 | 0 |
| DTCQ 4.6 | 0.161 | 0.005 | 35.731 | 0 |
| MAAQ 1.1 | 0.721 | 0.009 | 84.127 | 0 |
| MAAQ 1.2 | 0.479 | 0.009 | 54.676 | 0 |
| MAAQ 1.3 | 0.459 | 0.01 | 48.13 | 0 |
| MAAQ 1.4 | 0.71 | 0.008 | 85.682 | 0 |
| AEQ 1.1 | 0.437 | 0.007 | 60.109 | 0 |
| AEQ 1.2 | 0.406 | 0.007 | 54.133 | 0 |
| AEQ 1.3 | 0.377 | 0.006 | 66.75 | 0 |
| AEQ 2.10 | 0.602 | 0.007 | 81.685 | 0 |
| AEQ 2.11 | 0.901 | 0.01 | 87.348 | 0 |
| AEQ 2.12 | 0.27 | 0.007 | 40.507 | 0 |
| AEQ 2.13 | 0.686 | 0.009 | 74.017 | 0 |
| AEQ 2.14 | 0.98 | 0.01 | 94.093 | 0 |
| AEQ 3.15 | 0.678 | 0.008 | 80.549 | 0 |
| AEQ 3.16 | 0.878 | 0.011 | 76.918 | 0 |
| AEQ 3.17 | 0.366 | 0.007 | 52.737 | 0 |
| Factor 1 | 0.73 | 0.02 | 36.73 | 0 |
| Factor 2 | 0.797 | 0.012 | 64.727 | 0 |
| Factor 3 | 0.276 | 0.009 | 32.379 | 0 |
| Factor 4 | 0.56 | 0.009 | 60.324 | 0 |
| Factor 5 | 0.094 | 0.007 | 13.255 | 0 |

**Supplementary Table 14**: Confirmatory Factor Analysis Loadings with Cluster-Robust Standard Errors (Cluster = Participant ID)

| Variable | Estimate | Std.Err (cluster-robust) | Std. Loading | Factor |
| --- | --- | --- | --- | --- |
| DTCQ 1.1 | 1 | 0 | 0.84399754 | Factor 1 |
| DTCQ 1.2 | 0.98982521 | 0.09410049 | 0.82670134 | Factor 1 |
| DTCQ 1.3 | 1.0065969 | 0.0823429 | 0.84235667 | Factor 1 |
| DTCQ 1.4 | 0.95105027 | 0.08149516 | 0.80063495 | Factor 1 |
| DTCQ 2.5 | 1.0181503 | 0.04630736 | 0.85704524 | Factor 1 |
| DTCQ 2.6 | 1.03577668 | 0.04944959 | 0.87519909 | Factor 1 |
| DTCQ 3.1 | 1 | 0 | 0.89294617 | Factor 2 |
| DTCQ 3.2 | 0.86031805 | 0.02107245 | 0.76907986 | Factor 2 |
| DTCQ 3.3 | 0.97776599 | 0.04381297 | 0.86886349 | Factor 2 |
| DTCQ 3.4 | 0.97102284 | 0.04029502 | 0.86564809 | Factor 2 |
| DTCQ 4.5 | 0.92571456 | 0.02722806 | 0.82640477 | Factor 2 |
| DTCQ 4.6 | 1.02674714 | 0.02844307 | 0.91602304 | Factor 2 |
| MAAQ 1.1 | 1 | 0 | 0.52621336 | Factor 3 |
| MAAQ 1.2 | 1.36694269 | 0.08288337 | 0.71994856 | Factor 3 |
| MAAQ 1.3 | 1.39897024 | 0.12698644 | 0.73533383 | Factor 3 |
| MAAQ 1.4 | 1.03423894 | 0.11874136 | 0.54209699 | Factor 3 |
| AEQ 1.1 | 1 | 0 | 0.7494656 | Factor 4 |
| AEQ 1.2 | 1.03678865 | 0.03311187 | 0.77291729 | Factor 4 |
| AEQ 1.3 | 1.04988632 | 0.039921 | 0.78779024 | Factor 4 |
| AEQ 2.10 | 0.83516388 | 0.04437602 | 0.62717919 | Factor 4 |
| AEQ 2.11 | 1 | 0 | 0.30689464 | Factor 5 |
| AEQ 2.12 | 2.78392497 | 0.52879086 | 0.8538267 | Factor 5 |
| AEQ 2.13 | 1.7725134 | 0.22516657 | 0.547947 | Factor 5 |
| AEQ 2.14 | 0.46700467 | 0.16986022 | 0.14293616 | Factor 5 |
| AEQ 3.15 | 1.8498425 | 0.37306835 | 0.56674375 | Factor 5 |
| AEQ 3.16 | 1.04169721 | 0.13844366 | 0.3222102 | Factor 5 |
| AEQ 3.17 | 2.58988331 | 0.51256049 | 0.79497304 | Factor 5 |

**Supplementary Table 15**: Confirmatory Factor Analysis Loadings with Bootstrap Uncertainty (2,000 Draws)

| Variable | Estimate | Std.Err (bootstrap) | 95% CI (lower) | 95% CI (upper) | Std. Loading | Factor |
| --- | --- | --- | --- | --- | --- | --- |
| DTCQ 1.1 | 1 | 0 | 1 | 1 | 0.84399754 | Factor 1 |
| DTCQ 1.2 | 0.98982521 | 0.0150402 | 0.96076386 | 1.01998134 | 0.82670134 | Factor 1 |
| DTCQ 1.3 | 1.0065969 | 0.01318796 | 0.97974704 | 1.03145222 | 0.84235667 | Factor 1 |
| DTCQ 1.4 | 0.95105027 | 0.01344859 | 0.92450526 | 0.97727445 | 0.80063495 | Factor 1 |
| DTCQ 2.5 | 1.0181503 | 0.00894146 | 0.99945051 | 1.03512732 | 0.85704524 | Factor 1 |
| DTCQ 2.6 | 1.03577668 | 0.00829459 | 1.01879189 | 1.05139168 | 0.87519909 | Factor 1 |
| DTCQ 3.1 | 1 | 0 | 1 | 1 | 0.89294617 | Factor 2 |
| DTCQ 3.2 | 0.86031805 | 0.00492255 | 0.85063324 | 0.87010089 | 0.76907986 | Factor 2 |
| DTCQ 3.3 | 0.97776599 | 0.00793397 | 0.9624235 | 0.99336194 | 0.86886349 | Factor 2 |
| DTCQ 3.4 | 0.97102284 | 0.00769222 | 0.95560283 | 0.98606932 | 0.86564809 | Factor 2 |
| DTCQ 4.5 | 0.92571456 | 0.00569383 | 0.91448341 | 0.93689205 | 0.82640477 | Factor 2 |
| DTCQ 4.6 | 1.02674714 | 0.0062191 | 1.01438884 | 1.0386097 | 0.91602304 | Factor 2 |
| MAAQ 1.1 | 1 | 0 | 1 | 1 | 0.52621336 | Factor 3 |
| MAAQ 1.2 | 1.36694269 | 0.01881568 | 1.32954049 | 1.40318735 | 0.71994856 | Factor 3 |
| MAAQ 1.3 | 1.39897024 | 0.02854352 | 1.34342928 | 1.45381913 | 0.73533383 | Factor 3 |
| MAAQ 1.4 | 1.03423894 | 0.0251347 | 0.9840482 | 1.08384963 | 0.54209699 | Factor 3 |
| AEQ 1.1 | 1 | 0 | 1 | 1 | 0.7494656 | Factor 4 |
| AEQ 1.2 | 1.03678865 | 0.00790961 | 1.02091146 | 1.05171156 | 0.77291729 | Factor 4 |
| AEQ 1.3 | 1.04988632 | 0.0089237 | 1.0326291 | 1.06760539 | 0.78779024 | Factor 4 |
| AEQ 2.10 | 0.83516388 | 0.01036443 | 0.81499571 | 0.85588686 | 0.62717919 | Factor 4 |
| AEQ 2.11 | 1 | 0 | 1 | 1 | 0.30689464 | Factor 5 |
| AEQ 2.12 | 2.78392497 | 0.10758021 | 2.59387564 | 3.01444715 | 0.8538267 | Factor 5 |
| AEQ 2.13 | 1.7725134 | 0.04924088 | 1.68345427 | 1.87656469 | 0.547947 | Factor 5 |
| AEQ 2.14 | 0.46700467 | 0.03006447 | 0.41177996 | 0.53118608 | 0.14293616 | Factor 5 |
| AEQ 3.15 | 1.8498425 | 0.07267343 | 1.71986652 | 2.0039366 | 0.56674375 | Factor 5 |
| AEQ 3.16 | 1.04169721 | 0.0314622 | 0.98490823 | 1.1043608 | 0.3222102 | Factor 5 |
| AEQ 3.17 | 2.58988331 | 0.10316083 | 2.40933229 | 2.8087614 | 0.79497304 | Factor 5 |

**Supplementary Table 16**: Structural Equation Model Results (Year 5 → Year 6; MLR + FIML + Yuan–Bentler Robust Corrections)

(a) Robust model fit indices for the SEM estimated with maximum likelihood (MLR) and full-information maximum likelihood (FIML) missing-data handling, using Yuan–Bentler robust corrections (std.lv = TRUE; fixed.x = FALSE).

| **Metric** | **Value** |
| --- | --- |
| **N (cases)** | 664 |
| **Parameters** | 141 |
| **df** | 488 |
| **Scaled χ²** | 1064.649 |
| **Scaled χ² df** | 488.000 |
| **Scaled χ² p** | 0.000 |
| **CFI (robust)** | 0.883 |
| **TLI (robust)** | 0.866 |
| **RMSEA (robust)** | 0.069 |
| **RMSEA 90% CI (robust)** | [0.000, 0.046] |
| **SRMR** | 0.071 |

(b) Structural paths from latent factors (f₁–f₅) and covariates (visit age, SES, sex) to Year 6 outcomes, showing unstandardized estimates, robust standard errors, z-values, p-values, 95% CIs, and standardized (Std.all) coefficients.

** p < .05; ** p < .01; *** p < .001*

| **Outcome** | **Predictor** | **Estimate** | **Std.Err (robust)** | **z** | **p** | **95% CI** | **Std. Loading** |
| --- | --- | --- | --- | --- | --- | --- | --- |
| *Total Other Drugs Used* | Factor 1 | 0.014 | 0.068 | 0.21 | 0.834 | [-0.119, 0.148] | 0.014 |
| *Total Other Drugs Used* | Factor 2 | 0.049 | 0.105 | 0.46 | 0.643 | [-0.157, 0.254] | 0.049 |
| *Total Other Drugs Used* | Factor 3 | -0.085 | 0.148 | -0.58 | 0.563 | [-0.375, 0.204] | -0.086 |
| *Total Other Drugs Used* | Factor 4 | 0.384*** | 0.101 | 3.79 | **0.000** | [0.186, 0.583] | 0.385 |
| *Total Other Drugs Used* | Sex (M=1) | -0.007 | 0.191 | -0.04 | 0.972 | [-0.380, 0.367] | -0.003 |
| *Total Other Drugs Used* | Age | -0.006 | 0.064 | -0.09 | 0.926 | [-0.132, 0.120] | -0.006 |
| *Total Other Drugs Used* | SES | -0.090 | 0.12 | -0.75 | 0.452 | [-0.325, 0.145] | -0.091 |
| *Onset of Regular Drinking* | Factor 1 | -0.003 | 0.038 | -0.07 | 0.941 | [-0.077, 0.072] | -0.003 |
| *Onset of Regular Drinking* | Factor 3 | -0.052 | 0.053 | -0.99 | 0.322 | [-0.156, 0.051] | -0.053 |
| *Onset of Regular Drinking* | Factor 4 | 0.388*** | 0.047 | 8.28 | 0.0 | [0.296, 0.480] | 0.391 |
| *Onset of Regular Drinking* | Sex (M=1) | 0.027 | 0.075 | 0.36 | 0.717 | [-0.120, 0.174] | 0.014 |
| *Onset of Regular Drinking* | Age | 0.239*** | 0.038 | 6.28 | **0.000** | [0.164, 0.313] | 0.241 |
| *Onset of Regular Drinking* | SES | 0.060 | 0.039 | 1.54 | 0.124 | [-0.017, 0.137] | 0.061 |
| *Past Year Binge* | Factor 1 | -0.325 | 0.172 | -1.89 | 0.059 | [-0.663, 0.013] | -0.316 |
| *Past Year Binge* | Factor 2 | 0.130 | 0.104 | 1.26 | 0.209 | [-0.073, 0.333] | 0.126 |
| *Past Year Binge* | Factor 3 | -0.105 | 0.076 | -1.38 | 0.168 | [-0.254, 0.044] | -0.102 |
| *Past Year Binge* | Factor 4 | 0.246*** | 0.053 | 4.65 | **0.000** | [0.143, 0.350] | 0.239 |
| *Past Year Binge* | Sex (M=1) | 0.187* | 0.093 | 2.02 | **0.043** | [0.006, 0.369] | 0.091 |
| *Past Year Binge* | Age | -0.073 | 0.054 | -1.35 | 0.176 | [-0.179, 0.033] | -0.071 |
| *Past Year Binge* | SES | -0.016 | 0.065 | -0.24 | 0.807 | [-0.144, 0.112] | -0.016 |
| *Ever had sexual intercourse?* | Factor 1 | -0.105 | 0.054 | -1.93 | 0.053 | [-0.211, 0.001] | -0.104 |
| *Ever had sexual intercourse?* | Factor 4 | 0.170** | 0.058 | 2.93 | 0.003 | [0.056, 0.284] | 0.169 |
| *Ever had sexual intercourse?* | Sex (M=1) | -0.263* | 0.104 | -2.54 | 0.011 | [-0.466, -0.060] | -0.131 |
| *Ever had sexual intercourse?* | Age | 0.274*** | 0.058 | 4.74 | **0.000** | [0.161, 0.388] | 0.273 |
| *Ever had sexual intercourse?* | SES | -0.002 | 0.067 | -0.03 | 0.973 | [-0.134, 0.129] | -0.002 |

(c) Latent factor covariances, displaying unstandardized estimates, robust standard errors, z-values, p-values, and standardized correlations.

| **Left** | **Right** | **Covariance** | **Std.Err (robust)** | **z** | **p** | **Std. Loading** |
| --- | --- | --- | --- | --- | --- | --- |
| **Factor 1** | Factor 2 | 0.531 | 0.119 | 4.45 | 0.0 | 0.531 |
| **Factor 1** | Factor 3 | 0.074 | 0.065 | 1.13 | 0.256 | 0.074 |
| **Factor 1** | Factor 4 | -0.047 | 0.068 | -0.68 | 0.494 | -0.047 |
| **Factor 1** | Factor 5 | 0.105 | 0.082 | 1.27 | 0.205 | 0.105 |
| **Factor 2** | Factor 3 | 0.032 | 0.113 | 0.29 | 0.774 | 0.032 |
| **Factor 2** | Factor 4 | 0.018 | 0.097 | 0.18 | 0.856 | 0.018 |
| **Factor 2** | Factor 5 | 0.116 | 0.108 | 1.08 | 0.282 | 0.116 |
| **Factor 3** | Factor 4 | -0.169 | 0.07 | -2.42 | 0.015 | -0.169 |
| **Factor 3** | Factor 5 | -0.176 | 0.075 | -2.34 | 0.019 | -0.176 |
| **Factor 4** | Factor 5 | 0.727 | 0.036 | 19.94 | 0.0 | 0.727 |

(d) Standardized factor loadings for all observed indicators on their corresponding latent variables (f₁–f₅), estimated under the same model specification.

| **Variable** | **Estimate** | **Std.Err (robust)** | **Std. Loading** | **Factor** |
| --- | --- | --- | --- | --- |
| **DTCQ 1.1** | 0.863 | 0.084 | 0.865 | Factor 1 |
| **DTCQ 1.2** | 0.908 | 0.106 | 0.908 | Factor 1 |
| **DTCQ 1.3** | 0.905 | 0.114 | 0.905 | Factor 1 |
| **DTCQ 1.4** | 0.89 | 0.102 | 0.89 | Factor 1 |
| **DTCQ 2.5** | 0.923 | 0.108 | 0.923 | Factor 1 |
| **DTCQ 2.6** | 0.93 | 0.109 | 0.93 | Factor 1 |
| **DTCQ 3.1** | 0.917 | 0.085 | 0.883 | Factor 2 |
| **DTCQ 3.2** | 0.628 | 0.083 | 0.617 | Factor 2 |
| **DTCQ 3.3** | 0.945 | 0.123 | 0.908 | Factor 2 |
| **DTCQ 3.4** | 0.91 | 0.11 | 0.877 | Factor 2 |
| **DTCQ 4.5** | 0.818 | 0.094 | 0.793 | Factor 2 |
| **DTCQ 4.6** | 1.004 | 0.103 | 0.96 | Factor 2 |
| **MAAQ 1.1** | 0.548 | 0.058 | 0.548 | Factor 3 |
| **MAAQ 1.2** | 0.735 | 0.046 | 0.735 | Factor 3 |
| **MAAQ 1.3** | 0.705 | 0.052 | 0.706 | Factor 3 |
| **MAAQ 1.4** | 0.672 | 0.054 | 0.672 | Factor 3 |
| **AEQ 1.1** | 0.758 | 0.04 | 0.758 | Factor 4 |
| **AEQ 1.2** | 0.765 | 0.036 | 0.765 | Factor 4 |
| **AEQ 1.3** | 0.757 | 0.034 | 0.757 | Factor 4 |
| **AEQ 2.10** | 0.676 | 0.041 | 0.676 | Factor 4 |
| **AEQ 3.15** | 0.666 | 0.045 | 0.669 | Factor 4 |
| **AEQ 2.14** | 0.302 | 0.052 | 0.302 | Factor 4 |
| **AEQ 2.11** | 0.348 | 0.081 | 0.348 | Factor 5 |
| **AEQ 2.12** | 0.868 | 0.042 | 0.868 | Factor 5 |
| **AEQ 2.13** | 0.565 | 0.076 | 0.565 | Factor 5 |
| **AEQ 3.16** | 0.379 | 0.086 | 0.38 | Factor 5 |
| **AEQ 3.17** | 0.809 | 0.045 | 0.809 | Factor 5 |

**Supplementary Figure 1**: Timeline of Data Collection for SEM Variables


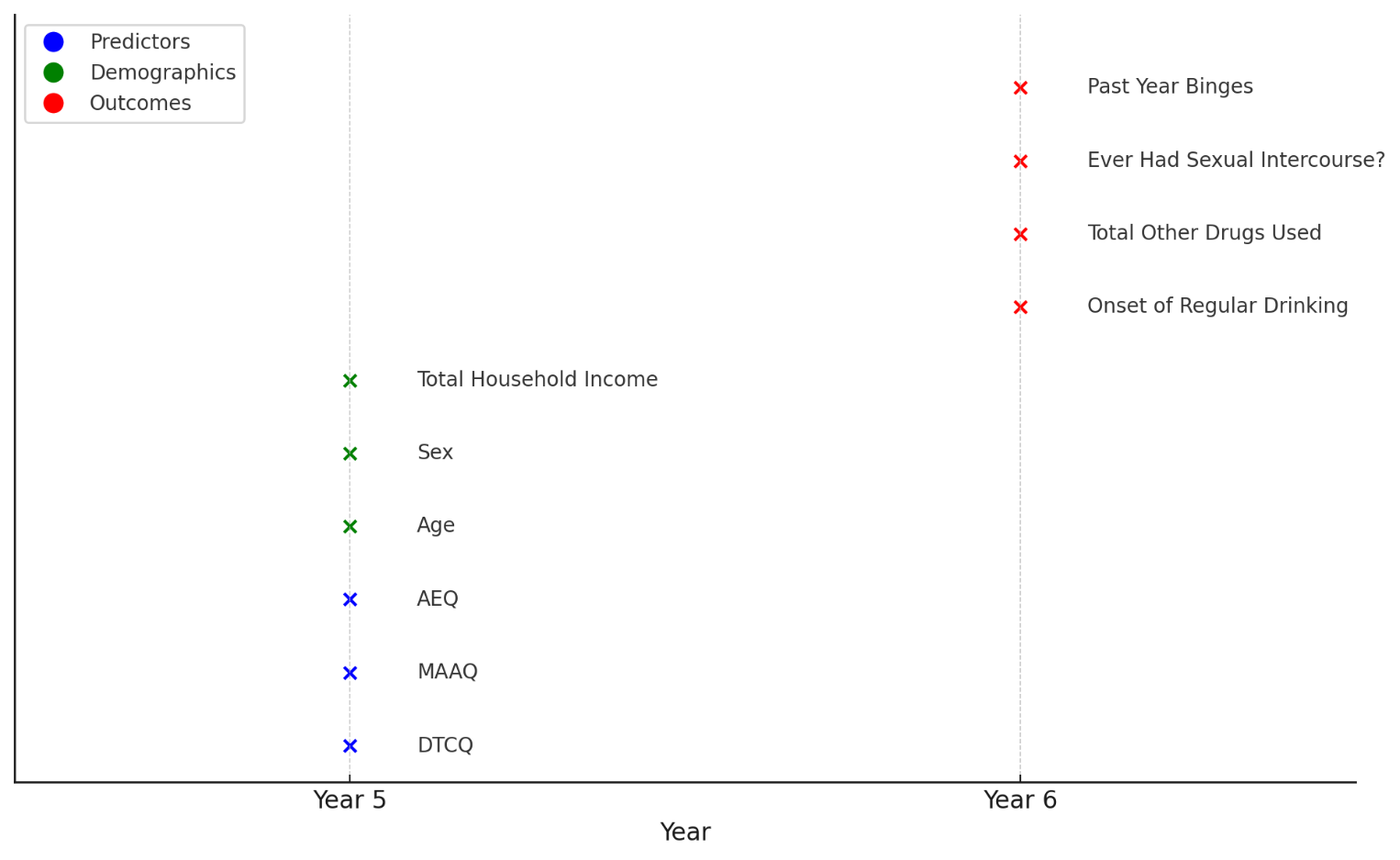


This figure depicts the timeline of data collection for each variable from the NCANDA study used in constructing the structural equation model, with variables color-coded by role in the model.

**Supplementary Figure 2a
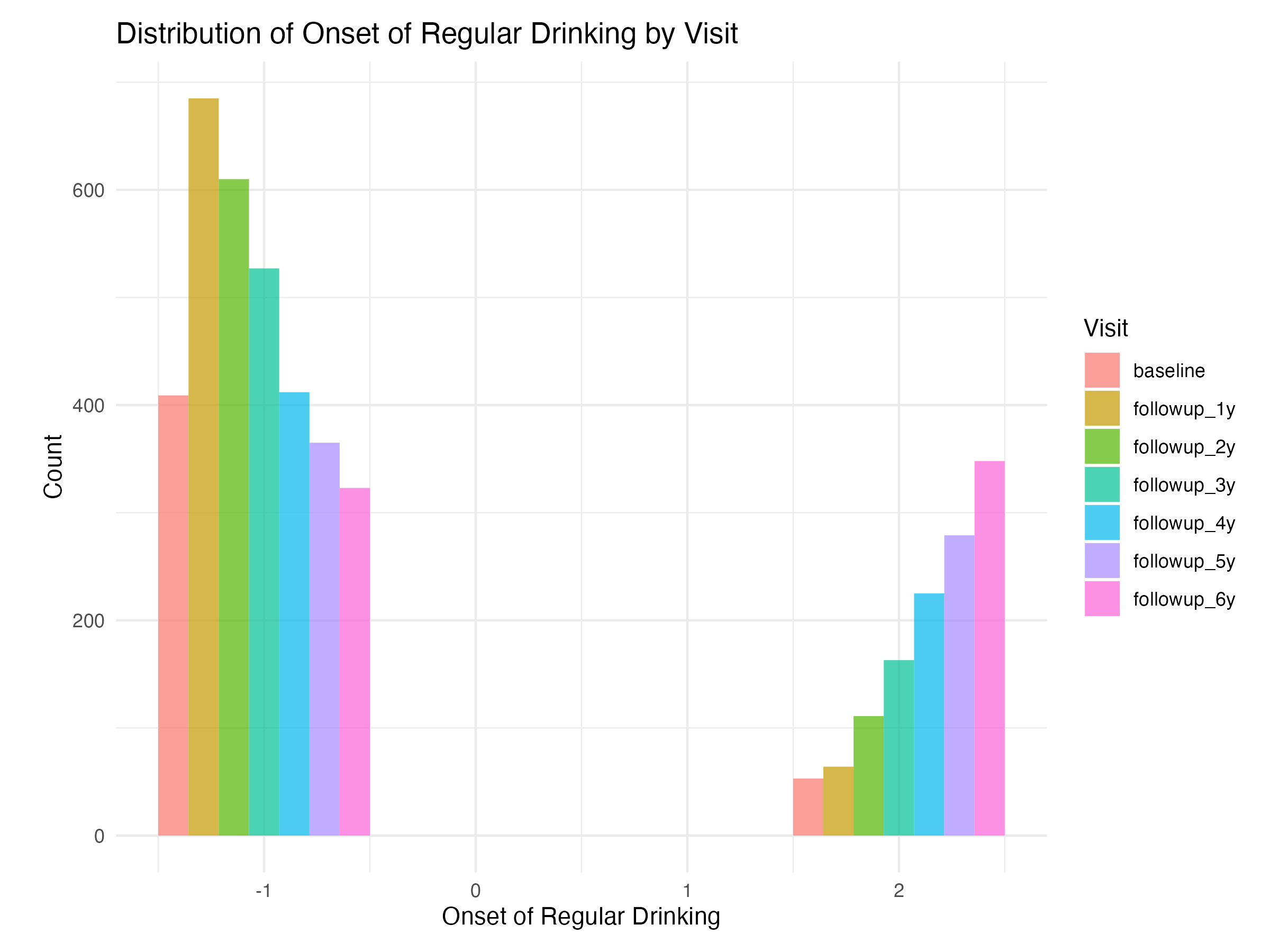
**

**Supplementary Figure 2b
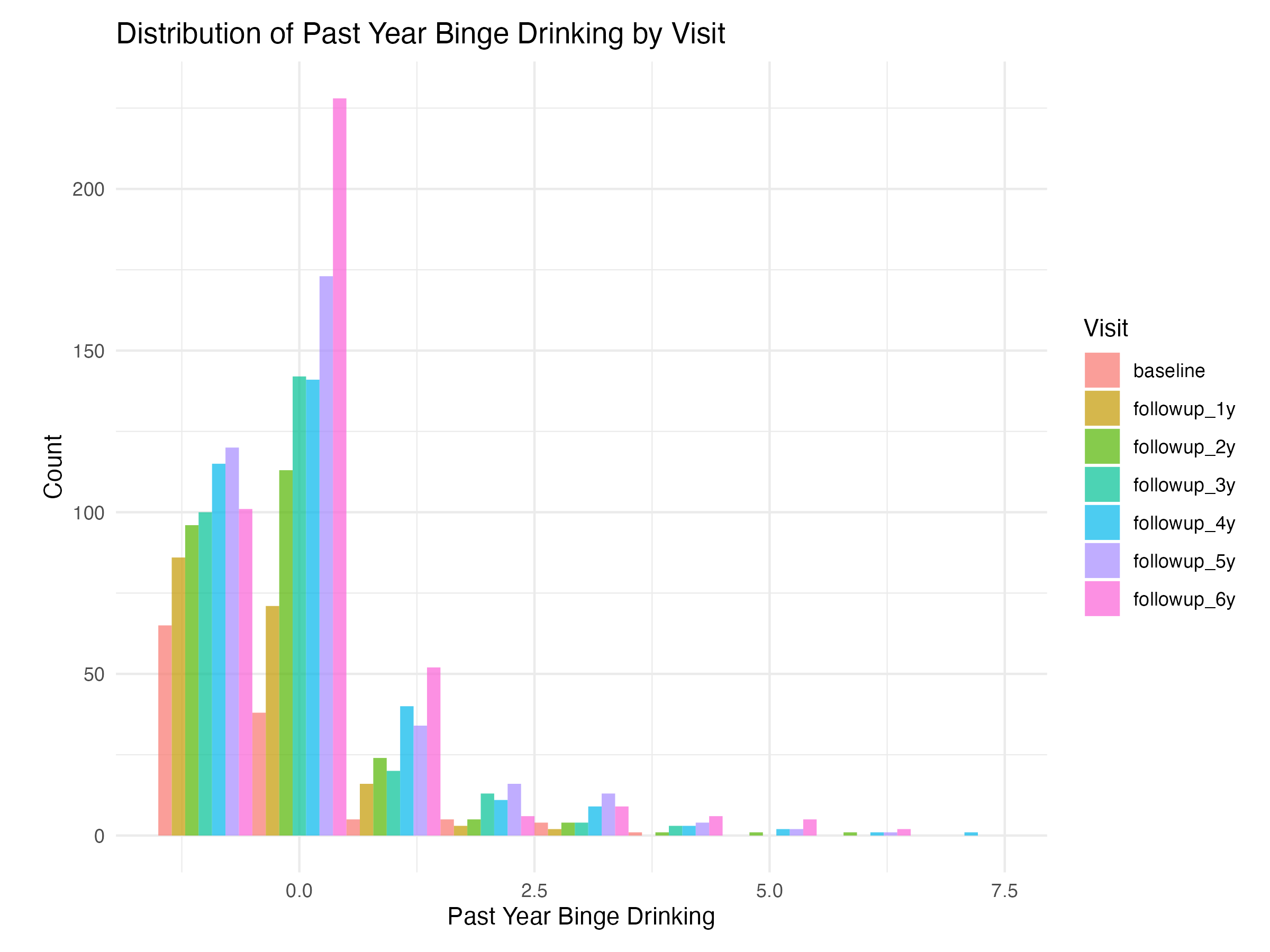
**

**Supplementary Figure 2c
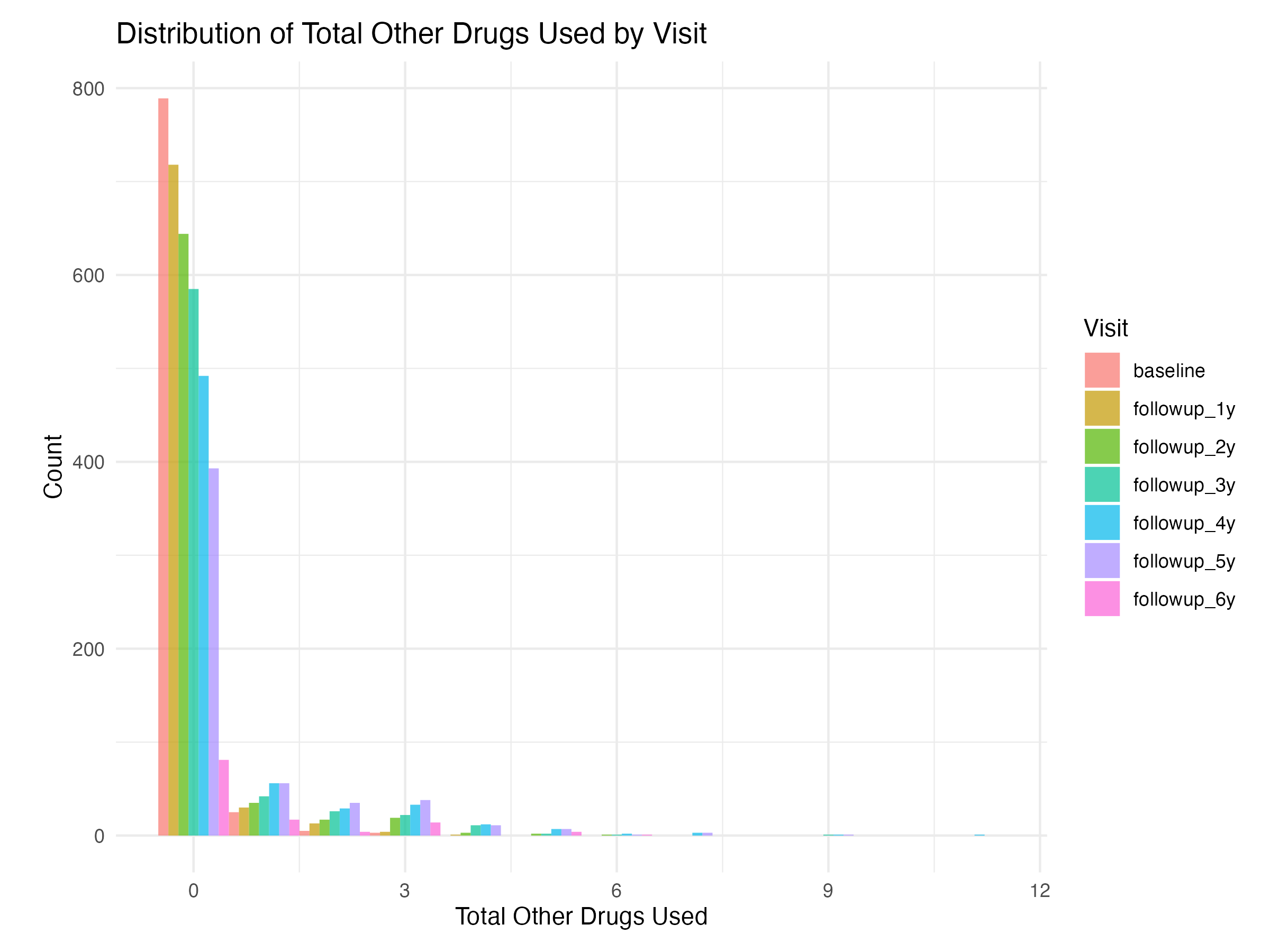
 Supplementary Figure 2d
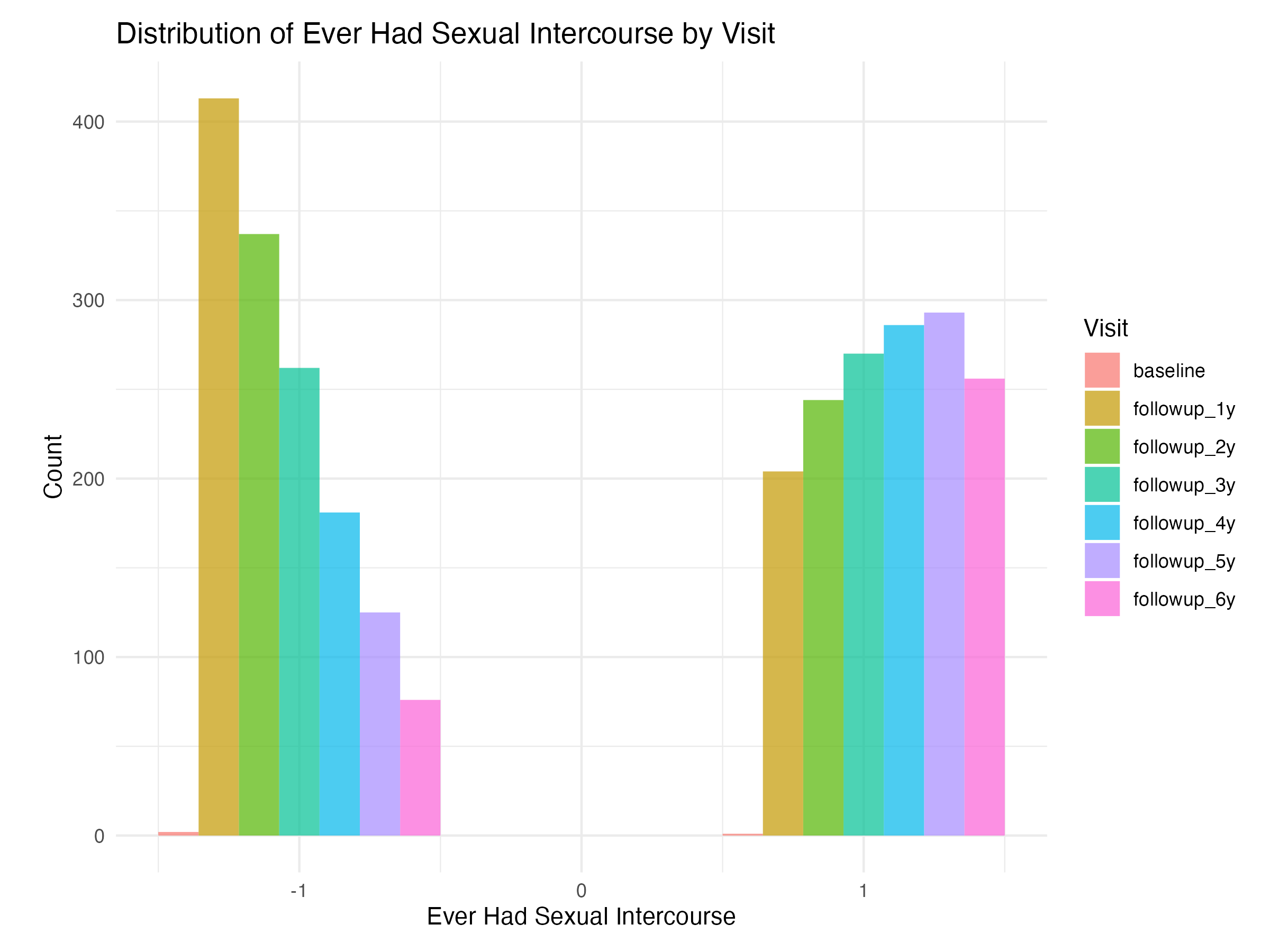
**

**Supplementary Figure 3a
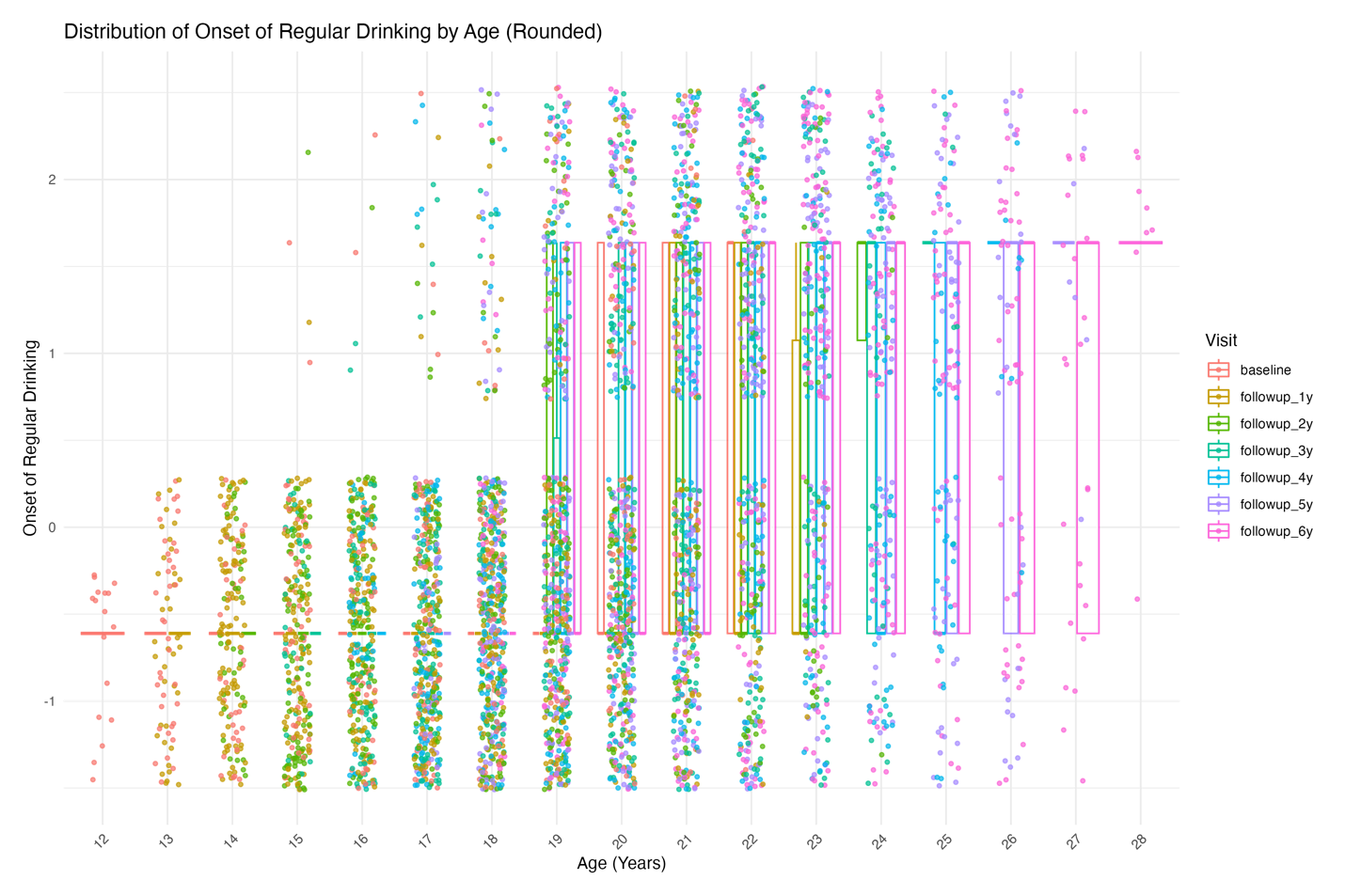
**

**Supplementary Figure 3b**

**
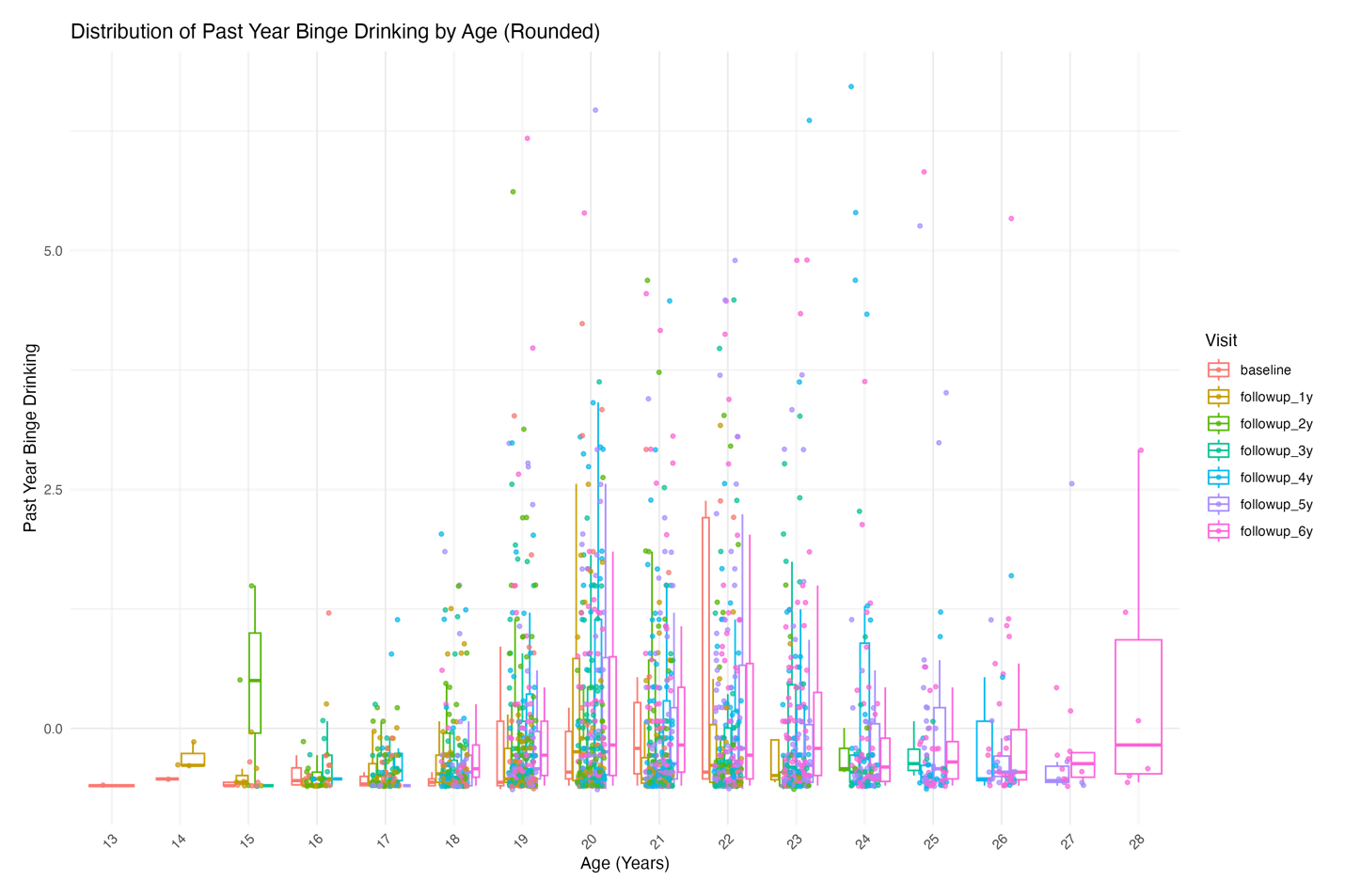
**

**Supplementary Figure 3c
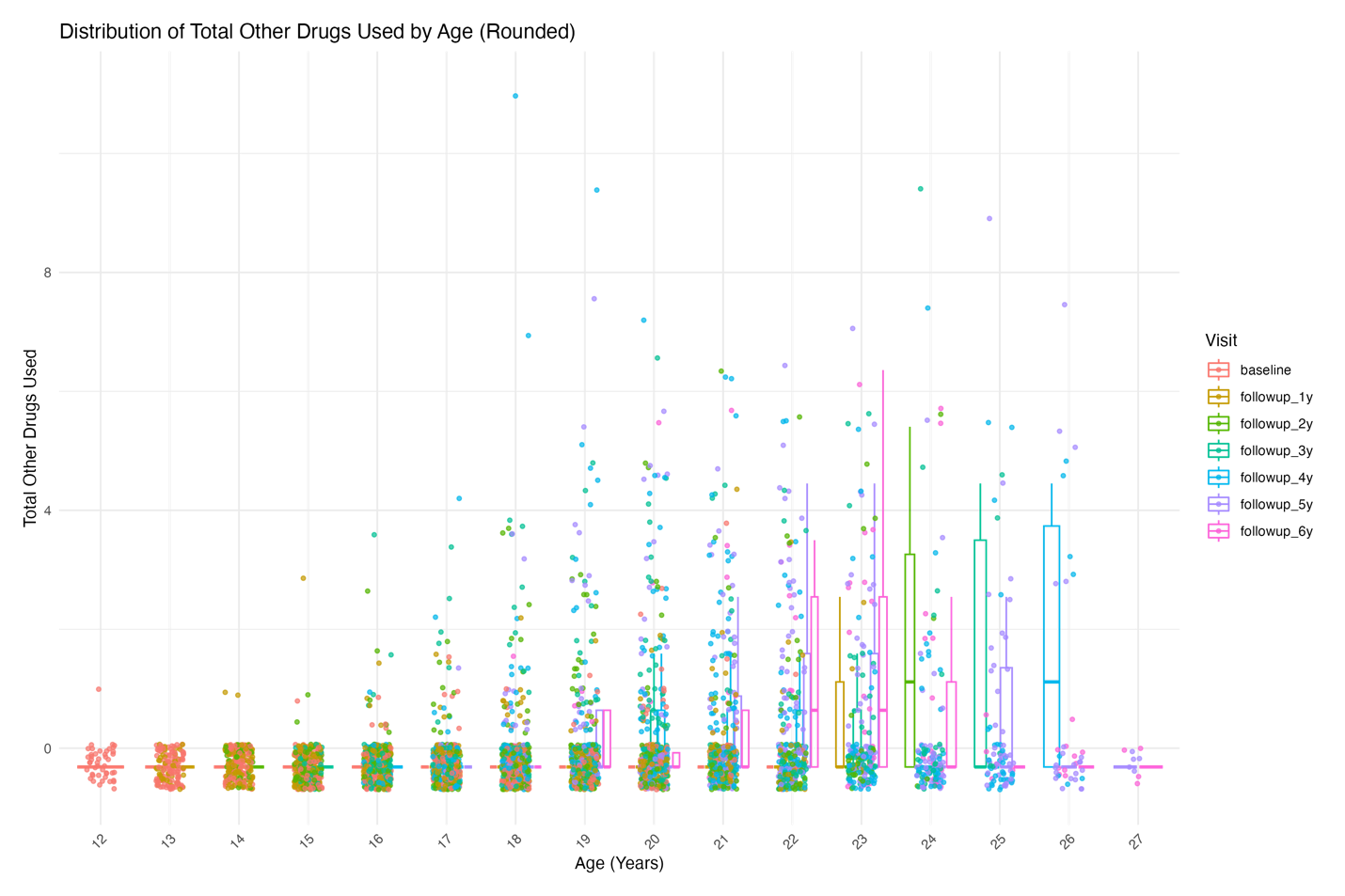
**

**Supplementary Figure 3d**

**
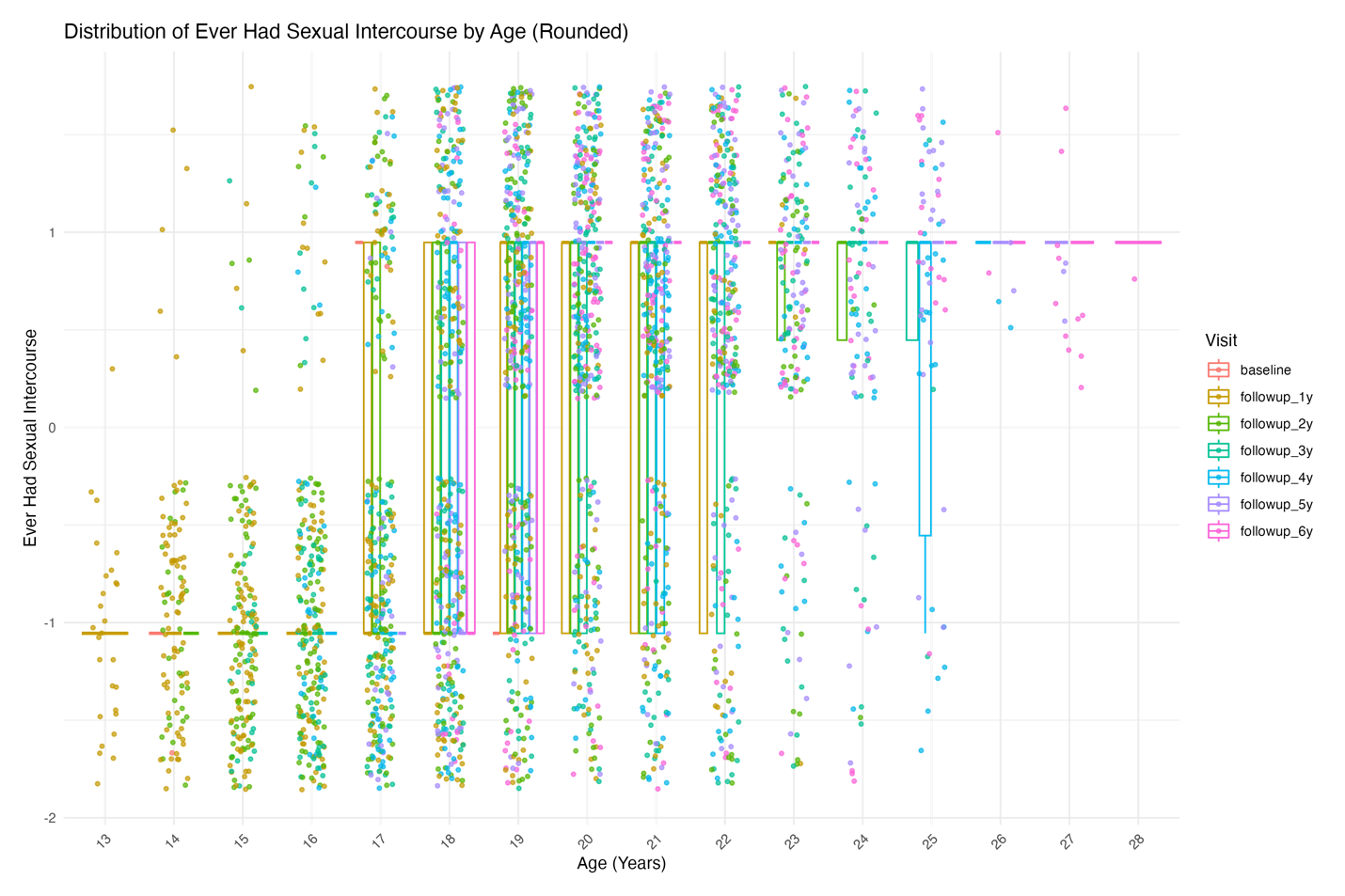
**

**Supplementary Description: DTCQ, MAAQ, and AEQ Question Prompts**

**DTCQ (Drug Taking Confidence Questionnaire)**

1.1 *If I were angry at the way things had turned out*

1.2 *If I had trouble sleeping*

1.3 *If I remembered something good that had happened*

1.4 *If I wanted to find out whether I could drink occasionally without getting hooked*
2.5 *If I unexpectedly found some booze or happened to see something that reminded me of drinking*
2.6 *If other people treated me unfairly or interfered with my plans*

3.1b *If I were angry at the way things had turned out*
3.2b *If I had trouble sleeping*
3.3b *If I remembered something good that had happened*
3.4b *If I wanted to find out whether I could use [drug used most frequently] occasionally without getting hooked*

4.5b *If I unexpectedly found some [drug used most frequently] or happened to see something that reminded me of using [drug used most frequently]*
4.6b *If other people treated me unfairly or interfered with my plans*

**MAAQ (Motives for Abstaining from Alcohol Questionnaire)**

1.1 *Alcohol may affect my studies.*

1.2 *My doctor told me not to drink alcohol.*

1.3 *My family disapproves of drinking.*

1.4 *Drinking alcohol is against my spiritual and religious beliefs.*

**AEQ (Alcohol Expectancy Questionnaire)**

1.1 *A person can talk with people of the opposite sex better after a few drinks of alcohol.*

1.2 *People feel more caring and giving after a few drinks of alcohol.*

1.3 *Drinking alcohol makes it easier to be with others and, in general, makes the world* 1.4 *seem like a nicer place.*

2.10 *Alcohol makes people feel more romantic.*
2.11 *Drinking alcohol causes hangovers.*
2.12 *Alcohol makes people more relaxed and less tense.*
2.13 *People laugh a lot and do silly or crazy things when they have been drinking.*
2.14 *People can control their anger better when they are drinking alcohol.*

3.15 *Alcoholic beverages make parties more fun.*
3.16 *After drinking alcohol, a person may lose control and run into things.*
3.17 *Drinking alcohol relaxes people.*
